# Novel Phenothiazine/Donepezil-like Hybrids Endowed with Antioxidant Activity for a Multi-Target Approach to the Therapy of Alzheimer’s Disease

**DOI:** 10.3390/antiox11091631

**Published:** 2022-08-23

**Authors:** Alessia Carocci, Alexia Barbarossa, Rosalba Leuci, Antonio Carrieri, Leonardo Brunetti, Antonio Laghezza, Marco Catto, Francesco Limongelli, Sílvia Chaves, Paolo Tortorella, Cosimo Damiano Altomare, Maria Amélia Santos, Fulvio Loiodice, Luca Piemontese

**Affiliations:** 1 Department of Pharmacy—Pharmaceutical Sciences, University of Bari Aldo Moro, via E. Orabona 4, 70125 Bari, Italy; 2 Centro de Química Estrutural, Institute of Molecular Sciences, Departamento de Engenharia Química, Instituto Superior Técnico, Universidade de Lisboa, Av. Rovisco Pais 1, 1049-001 Lisboa, Portugal

**Keywords:** Alzheimer’s disease, neurodegeneration, antioxidants, multi-functional drugs, donepezil, phenothiazine, FAAH, acetylcholinesterase, butyrylcholinesterase, Aβ

## Abstract

Alzheimer’s disease (AD) is a complex multi-factorial neurodegenerative disorder for which only few drugs (including donepezil, DPZ) are available as symptomatic treatments; thus, researchers are focusing on the development of innovative multi-target directed ligands (MTDLs), which could also alter the course of the disease. Among other pathological factors, oxidative stress has emerged as an important factor in AD that could affect several pathways involved in the onset and progression of the pathology. Herein, we propose a new series of hybrid molecules obtained by linking a phenothiazine moiety, known for its antioxidant properties, with *N*-benzylpiperidine or *N*-benzylpiperazine fragments, mimicking the core substructure of DPZ. The investigation of the resulting hybrids showed, in addition to their antioxidant properties, their activity against some AD-related targets, such as the inhibition of cholinesterases (both AChE and BChE) and in vitro Aβ_1-40_ aggregation, as well as the inhibition of the innovative target fatty acid amide hydrolase (FAAH). Furthermore, the drug-likeness properties of these compounds were assessed using cheminformatic tools. Compounds **11d** and **12d** showed the most interesting multi-target profiles, with all the assayed activities in the low micromolar range. In silico docking calculations supported the obtained results. Compound **13**, on the other hand, while inactive in the DPPH assay, showed the best results in the in vitro antioxidant cell assays conducted on both HepG2 and SHSY-5Y cell lines. These results, paired with the low or absent cytotoxicity of these compounds at tested concentrations, allow us to aim our future research at the study of novel and effective drugs and pro-drugs with similar structural characteristics.

## 1. Introduction

Alzheimer’s disease (AD) is characterized by a progressive loss of cognitive function due to a degeneration of the synapses, with evident neuronal damage and death, which results in a net decrease in the volume of some areas of the brain, in particular the hippocampus and parts of the cortex [1].

Despite the widely recognized multi-factorial profile of this disease, its etiology seems to be particularly linked to chronic oxidative stress, which can lead to tissue inflammation and cell death [2]. In fact, oxidative stress, caused by an imbalance between the production of ROS (Reactive Oxygen Species) and the natural antioxidant defenses of organisms such as scavengers or antioxidant enzymes, can cause DNA mutations, lipid peroxidation in the cell membrane, alterations and damage to cellular organelles, chiefly the mitochondria, and it has been linked to the pathogenesis of numerous conditions such as cancer [3], cardiovascular diseases (e.g., atherosclerosis and ischemia-reperfusion injury) [4], degenerating myopathies [5] and, in general, neurodegenerative diseases (NDs). While oxidative stress is not the only reason for the onset and progression of NDs, it is certainly an important link in the chain of events that lead to neuron loss [6].

On the other hand, the deposition of amyloid (senile) plaques in the brain is an important pathological hallmark correlate of AD. This phenomenon could be responsible for the initial damage to neurons and it could drive further neurodegeneration. Cholinergic nuclei are particularly vulnerable to these processes, and their degeneration, resulting in lower levels of neurotransmitter acetylcholine, is correlated with the progression of symptoms. Thus, the inhibition of both acetyl- and butyryl-cholinesterases (AChE and BChE, respectively) is a well-established therapeutic option [7]. Clinical observation showed other peculiar characteristics such as intra-cellular neurofibrillary tangles of hyperphosphorylated tau protein [8].

Interestingly, neurodegeneration is often linked to a dysregulation of inflammatory pathways, such as NRF2-dependent signaling, resulting in inflammation and oxidative stress [9]. Metal dyshomeostasis, which is directly correlated to neurotoxicity because of high levels of copper and zinc ions, can accelerate the formation of amyloid aggregates, and is related to oxidative damage as well. Indeed, redox-active metal ions (mainly Cu and Fe) can influence, directly or indirectly, the balance in oxidative reactions, generating ROS [10].

Currently, established therapies are essentially aimed at delaying the progression of symptoms related to the disease, mainly the slow and unstoppable memory loss. Only four drugs are available, including three cholinesterase inhibitors (donepezil, rivastigmine and galantamine) and an NMDA receptor blocker (memantine) [7]. Recently, FDA (Food and Drug Administration, US) granted “accelerated approval” to the clinical use of monoclonal antibody aducanumab [11]. This antibody, directed against amyloid-beta oligomers, is the first known therapeutic agent to actually alter disease progression and not merely delay it, and it will be administered in the USA to patients with severe AD. However, there is still uncertainty about the clinical benefits of aducanumab, and further post-approval trials are under way in the USA [11]. At the same time, the European Medicinal Agency (EMA) recommended refusing marketing authorization for this drug. Interestingly, the applicant company requested a re-examination of this recommendation, but subsequently withdrew it [12].

It is now recognized that, given the multi-factorial origin of AD, its therapy should be broadened to multiple pathological targets. For this reason, over the past twenty years, research focus in this field has shifted to the development of multi-functional therapeutic agents. Moreover, the limited success achieved by using hybrid compounds aimed at classical targets has also put the spotlight on the search for new therapeutic targets. The workflow for research of this type can involve the re-purpose of drugs, by designing molecules that bear a portion of a known drug (e.g., tacrine or donepezil), linked to fragments that have other pharmacological properties [7].

Following this paradigm, one recent work of ours [13] involved the design, synthesis and biological evaluation of a series of donepezil-like hybrids as inhibitors of three different enzymes, namely AChE, BChE and fatty acid amide hydrolase (FAAH). In silico studies were also performed to rationalize the obtained results and compounds were also assayed for their ability to inhibit amyloid aggregation and for their ability to chelate copper(II). These compounds included a donepezil-like *N*-benzylpiperidine or *N*-benzylpiperazine moiety in their structure and resulted as promising multi-functional compounds, as well as potent cholinesterase inhibitors [13]. This work is one of the first in which a particular cluster of biological properties was highlighted. In fact, together with inhibiting cholinesterases, these compounds showed to be capable of intervening in the delicate mechanisms of the endocannabinoid system (ECS) [14]. The ECS plays an important role in multiple processes in the central nervous system, including learning and memory, and its dysregulation is suspected to be one of the possible causes in the multi-factorial etiopathogenesis of AD. FAAH is involved in the degradation of the important endocannabinoid mediator anandamide and its expression is heightened in inflammatory states and during neurodegenerative processes. Its inhibition may therefore be a feasible route in the search for new therapeutic options for the treatment of AD [15].

In other recent papers [16,17] our research group was able to obtain nature-inspired structures, which were, in some cases, able to have evident antioxidant effects, but once they were linked to the *N*-benzylpiperazine moiety, this activity was lost.

**Figure 1:**
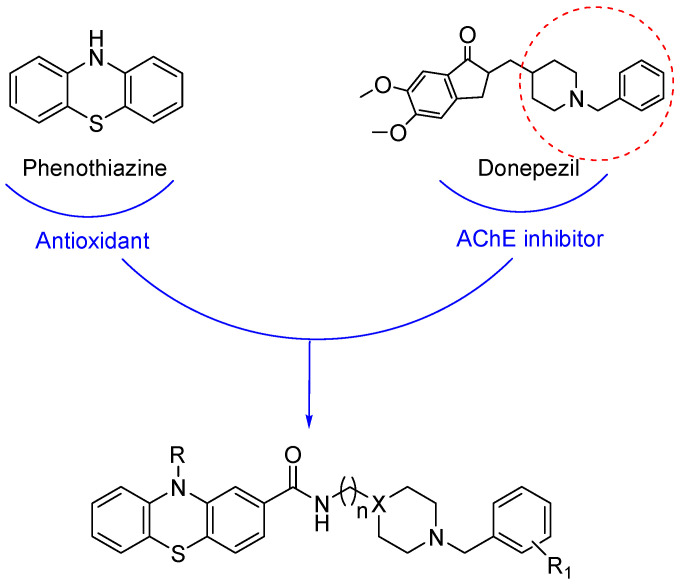
Drug design for the hybrids **11a–e**, **12a–e** and **13**.

In the present work, we designed and prepared a series of eleven derivatives (Figure 1) with the aim of combining for the first time five different activities in one molecule. As in the recent past, we decided to use both *N*-benzylpiperidine and *N*-benzylpiperazine as donepezil-like moieties, using different substitutions on the aromatic ring, on the basis of the results obtained in another of our recent publications [18]. These moieties were linked via the classic one- or two-methylene-amido chains [13,17,18,19,20] to the phenothiazine fragment. This nucleus is known as a very effective antioxidant [21] and also as an inhibitor of the tau protein aggregation in neurons, thus being the active moiety of Rember^®^ (methylthioninium chloride), a TauRx drug that entered phase III clinical evaluation in March 2010 [22], and was recently included in several hybrids containing the tacrine moiety in a preliminary study of novel anti-Alzheimer’s disease agents [23,24].

Herein, we studied a different kind of substitution, which could allow to maintain the antioxidant properties of the obtained hybrids. Besides the inhibition of cholinesterases and amyloid aggregation, we again explored FAAH among the panel of targets, and we deeply studied the antioxidant properties and cytotoxicity of these compounds.

## 2. Materials and Methods

### 2.1. Chemistry

Chemicals were used without any further purification and purchased from common suppliers. Geduran silica gel 60A was generally used as stationary phase for column chromatography, where necessary. A HPMS 6890-5973 MSD spectrometer was used for mass spectrometry analyses equipped with a HP ChemStation or with an Agilent LC–MS 1100 Series. For exact mass analyses, in particular, we employed an LC–MSD Trap System VL spectrometer equipped with electrospray ionization (ESI). Nuclear Magnetic Resonance (NMR) spectra were recorded in the specific deuterated solvent (as indicated in each procedure) using Agilent VNMRS500 or Varian Mercury 300 NMR instruments. Chemical shifts (*δ*) are reported as parts per million (ppm) and coupling constants (J) in Hertz (Hz). A selection of spectra was reported in the Supplementary Material File (Figure S1). Melting points of solid compounds (uncorrected) were determined in open capillaries on a Gallenkamp electrothermal apparatus. The purity of all tested compounds, based on the panel of analyses performed was estimated as >95%.

#### 2.1.1. Preparation of 1-(10*H*-phenothiazin-10-yl)ethenone (**1**)

Acetyl chloride (15.0 mmol, 1.5 eq) was added dropwise to a stirred suspension of ZnCl_2_ (15.0 mmol, 1.5 eq) in CH_2_Cl_2_ (40 mL) cooled in an ice bath. The mixture was stirred at 0 °C for 20 min, and then the ice bath was removed, and 10*H*-phenothiazine was added (10.0 mmol, 1 eq). The mixture was stirred for 24 h at room temperature. Then, the reaction mixture was diluted with ice-cold H_2_O (40 mL) and extracted with CH_2_Cl_2_. The collected organic layers were dried with anhydrous Na_2_SO_4_, filtered and concentrated under reduced pressure, obtaining a green solid, yield; 98%, m.p. = 202–204 °C. ^1^H-NMR (300 MHz, CDCl_3_) *δ* (ppm): 2.20 (s, 3H, CH_3_), 7.19–7.29, 7.30–7.35 and 7.42–7.51 (m, 8H, aromatics); ^13^C-NMR (75 MHz, CDCl_3_) *δ* (ppm): 23.3, 127.0, 127.2, 127.4, 128.2, 133.3, 139.2, 169.6. GS-MS *m*/*z* (%): 241 (25) [M]^+^, 199 (100). Other spectroscopic data are in agreement with the literature [25].

#### 2.1.2. Preparation of 1,1′-(10*H*-phenothiazine-2,10-diyl)diethanone (**2**)

Acetyl chloride (18.6 mmol, 3 eq) was slowly added dropwise under inert atmosphere to a stirred solution of 1-(10*H*-phenothiazin-10-yl)ethenone (**1**, 6.2 mmol, 1 eq) and AlCl_3_ (24.8 mmol, 4 eq) in anhydrous CH_2_Cl_2_ (35 mL) cooled in an ice bath. The reaction mixture was stirred at 50 °C for 6 h and at room temperature for further 15 h. Then, ice-cold H_2_O was slowly added, and the organic solvent was removed under reduced pressure. The resulting aqueous layer was extracted with ethyl acetate and the organic portion was washed with 6 N HCl and NaHCO_3_ saturated solution. The organic portion was dried over anhydrous Na_2_SO_4_, filtered and concentrated at reduced pressure. The crude brown solid was purified by column chromatography (*n*-hexane/EtOAc 7:3), affording a glassy solid, yield 37%. ^1^H-NMR (300 MHz, CDCl_3_) *δ* (ppm): 2.21 (s, 3H, NCOCH_3_), 2.60 (s, 3H, COCH_3_), 7.22–7.52 (m, 5H, aromatics), 7.81 (dd, J = 8.1 Hz, 1.8 Hz, 1H, aromatic), 8.09 (d, J = 1.5 Hz, 1H, aromatic); ^13^C-NMR (75 MHz, CDCl_3_) *δ* (ppm): 23.2, 26.9, 126.6, 127.4, 127.7, 128.1, 132.3, 136.2, 138.7, 139.1, 139.3. GS-MS *m*/*z* (%): 283 (15) [M]^+^, 241 (100) [26].

#### 2.1.3. Preparation of 10-acetyl-10*H*-phenothiazine-2-carboxylic Acid (**3**) and 10*H*-phenothiazine-2-carboxylic Acid (**4**)

Sublimated iodine (3.3 mmol, 1 eq) was added to a solution of 1,1′-(10*H*-phenothiazine-2,10-diyl)diethanone (**2**, 3.3 mmol, 1 eq) in pyridine (12 mL), and the reaction mixture was stirred at 110 °C for 15 min and at room temperature for 20 h. Then, solvent was removed *in vacuo*, obtaining a crude, which was purified by column chromatography (*n*-hexane/EtOAc 7:3), affording product **3**. 2 N NaOH (16 mmol, 4.8 eq), was subsequently added and the reaction was stirred at 100 °C for 2 h; then, after cooling down, the mixture was washed with ethyl acetate. The aqueous portion was acidified with 6 N HCl and extracted with ethyl acetate. The collected organic portions were washed with water, dried over anhydrous Na_2_SO_4_, filtered and concentrated at reduced pressure, to give a crude, which was purified by column chromatography (*n*-hexane/EtOAc 7:3), obtaining compound 4.

##### 10-Acetyl-10*H*-phenothiazine-2-carboxylic Acid (**3**)

The title compound was obtained as a green solid, yield 70%. ^1^H-NMR (300 MHz, CD_3_OD), *δ* (ppm): 2.25 (s, 3H, CH_3_), 7.20–7.50 and 7.33–7.40 (m, 7H, aromatics). HRMS (C_15_H_11_NO_3_S-H^–^): calculated: 284.0387; found: 284.0386.

##### 10*H*-phenothiazine-2-carboxylic Acid (**4**)

The title compound was obtained as a brown solid, yield 70%, m.p. = 249–251 °C. ^1^H-NMR (300 MHz, CDCl_3_), *δ* (ppm): 6.59–6.62, 6.68–6.78, 6.83–6.97 and 7.90–8.00 (m, 7H, aromatics), 8.25 (s, COOH). ^13^C NMR (75 MHz, CDCl_3_) *δ* (ppm): 114.6, 114.9, 116.7, 122.2, 123.3, 124.5, 126.0, 126.3, 127.7, 129.7, 142.6, 168.8. HRMS (C_13_H_9_NO_2_S-H^–^): calculated: 242.0281; found: 242.0278.

#### 2.1.4. Preparation of 2-(piperidin-4-ylmethyl)isoindoline-1,3-dione (**5**)

An intimate mixture of 4-(aminomethyl)piperidine (10 mmol, 1 eq) and phthalic anhydride (powder, 10 mmol, 1 eq) was left at 150 °C for 4 h. The resulting solid was added with a solution of HCl in absolute ethanol solution (1 N, 8 mL). After 10 min, the solvent was removed under vacuum. The title compound was obtained as a white solid (hydrochloride salt) after crystallization with absolute ethanol, yield 25%; m.p. = 214 °C dec. ^1^H-NMR (300 MHz, DMSO-d_6_) *δ* (ppm): 1.37–1.46, 1.73–2.01 and 2.70–3.28 (m, 9H, piperidine), 3.47 (d, 2H, NCH_2_CH), 7.83–7.91 (m, 4H aromatics). ESI-MS *m*/*z*: (IP: positive) 249 [M + H]^+^ [13].

#### 2.1.5. General Procedure for the Preparation of 2-((1-benzylpiperidin-4-yl)methyl)isoindoline-1,3-dione Derivatives (**7a–e**)

Compound **5** (2.5 mmol, 1 eq) was dissolved in 96° ethanol (12 mL) and added with triethylamine (5 mmol, 2 eq) and the appropriate benzyl bromide (2.5 mmol, 1 eq). The mixture was stirred for 24 h at RT. Subsequently, the solvent was removed *in vacuo* and the resulting residue was partitioned between distilled water and dichloromethane. The aqueous layer was then extracted twice with CH_2_Cl_2_ and the collected organic portions were dried over anhydrous sodium sulfate and filtered. After concentration under reduced pressure, the obtained crude solids were purified by chromatography column in different conditions.

##### 2-((1-Benzylpiperidin-4-yl)methyl)isoindoline-1,3-dione (**7a**)

Starting from benzyl bromide, eluent: EtOAc/MeOH 97:3. The title compound was obtained as a white solid, yield 41%; m.p. = 135–136 °C. ^1^H-NMR (300 MHz, DMSO-d_6_) *δ* (ppm): 1.07–1.15, 1.58–1.76 and 3.44–3.50 (m, 9H, piperidine), 2.73 (d, 2H, NCH_2_CH), 3.41 (s, 2H, NCH_2_Ph), 7.15–7.40 (m, 5H, aromatics, Ph–CH_2_), 7.79–7.91 (m, 4H, phthalimide). ESI-MS *m*/*z*: (IP: positive) 335 [M + H]^+^ [13].

##### 2-((1-(2-Chlorobenzyl)piperidin-4-yl)methyl)isoindoline-1,3-dione (**7b**)

Starting from 2-chlorobenzyl bromide, eluent: 100% EtOAc. The title compound was obtained as a white solid, yield 53%; m.p. = 109–111 °C. ^1^H-NMR (300 MHz, CDCl_3_) *δ* (ppm): 1.35–1.48, 1.58–1.69, 1.74–1.86, 1.96–2.11 and 2.84–2.93 (m, 9H, piperidine), 3.53–3.64 (m, 4H, 2H NCH_2_Ph + 2H NCH_2_CH), 7.11–7.22, 7.29–7.32 and 7.43–7.51 (m, 4H, Ph–CH_2_), 7.67–7.74 and 7.77–7.87 (m, 4H, phtalimide); ^13^C-NMR (75 MHz, CDCl_3_) *δ* (ppm): 30.9, 35.3, 43.3, 53.3, 59.3, 123.2, 126.5, 127.9, 129.2, 130.4, 132.0, 133.9, 168.6. GS-MS *m*/*z* (%): 368 (13) [M]^+^, 206 (100).

##### 2-((1-(2-Methylbenzyl)piperidin-4-yl)methyl)isoindoline-1,3-dione (**7c**)

Starting from 2-methylbenzyl bromide, eluent: 100% EtOAc. The title compound was obtained as a white solid, yield 78%; m.p. = 112–114 °C. ^1^H-NMR (300 MHz, CDCl_3_) *δ* (ppm): 1.30–1.41, 1.58–1.68, 1.74–1.85, 1.90–1.98 and 2.80–2.90 (m, 9H, piperidine), 2.33 (s, 3H, CH_3_), 3.41 (s, 2H, NCH_2_Ph), 3.59 (d, 2H, J = 7.2 Hz, NCH_2_CH), 7.10–7.20 and 7.25–7.28 (m, 4H, Ph–CH_2_), 7.65–7.75 and 7.81–7.86 (m, 4H, phtalimide); ^13^C-NMR (75 MHz, CDCl_3_) *δ* (ppm): 19.5, 30.3, 35.6, 43.6, 53.4, 61.1, 123.4, 125.5, 129.7, 130.3, 132.2, 134.0, 137.5, 168.8. ESI-MS *m*/*z*: (IP: positive) 349 [M + H]^+^.

##### 2-((1-(2-Fluorobenzyl)piperidin-4-yl)methyl)isoindoline-1,3-dione (**7d**)

Starting from 2-fluorobenzyl bromide, eluent: 100% EtOAc. The title compound was obtained as a white solid, yield 69%; m.p. = 109–111 °C. ^1^H-NMR (300 MHz, CDCl_3_) *δ* (ppm): 1.35–1.47, 1.60–1.68, 1.73–1.83, 1.97–2.06 and 2.87–2.93 (m, 9H, piperidine), 3.56–3.60 (m, 4H, 2H NCH_2_Ph + 2H NCH_2_CH), 6.97–7.02, 7.05–7.11, 7.18–7.25 and 7.35–7.40 (m, 4H, Ph–CH_2_), 7.66–7.73 and 7.80–7.86 (m, 4H, phtalimide); ^13^C-NMR (75 MHz, CDCl_3_) *δ* (ppm): 28.8, 35.3, 43.3, 52.8, 55.2, 115.2, 123.1, 123.8, 132.0, 133.9, 168.6. GS-MS *m*/*z* (%): 352 (9) [M]^+^, 109 (100).

##### 2-((1-(3-Methoxybenzyl)piperidin-4-yl)methyl)isoindoline-1,3-dione (**7e**)

Starting from 3-methoxybenzyl bromide, eluent: 100% EtOAc. The title compound was obtained as a pale-yellow solid, yield 30%. ^1^H-NMR (500 MHz, CDCl_3_) *δ* (ppm): 1.33–1.43, 1.58–1.65, 1.73–1.82, 1.86–1.95 and 2.79–2.89 (m, 9H, piperidine), 3.44 (s, 2H, NCH_2_Ph), 3.58 (d, 2H, J = 7.1 Hz, NCH_2_CH), 3.79 (s, 3H, OCH_3_), 6.73–6.78, 6.83–6.89 and 7.16–7.21 (m, 4H, Ph–CH_2_), 7.66–7.72 and 7.79–7.85 (m, 4H, phtalimide). GS-MS *m*/*z* (%): 364 (10) [M]^+^, 243 (93), 202 (53), 121 (100).

##### General Procedure for the Preparation of 2-(2-(4-Benzylpiperazin-1-yl)ethyl)isoindoline-1,3-dione Derivatives (**8a–e**)

An intimate mixture of 1-(2-aminoethyl)-piperazine (10 mmol, 1 eq) and phthalic anhydride (powder, 10 mmol, 1 eq) was left at 160 °C for 4 h. The so obtained dark oil was dissolved without any purification in 96° ethanol (20 mL) and added with KOH (12 mmol, 1.2 eq) and the specific benzyl bromide (10 mmol, 1 eq). The mixture was stirred for 24 h at RT. Subsequently, the solvent was removed *in vacuo* and the resulting residue was partitioned between distilled water and diethyl ether. The aqueous layer was then extracted twice with Et_2_O and the collected organic portions were washed with brine, dried over anhydrous sodium sulfate and filtered. After concentration under reduced pressure, the crude products were purified by column chromatography in various conditions, except for **8d**, which was used in the next step as it was.

##### 2-(2-(4-Benzylpiperazin-1-yl)ethyl)isoindoline-1,3-dione (**8a**)

Starting from benzyl bromide, eluent: *n*-hexane/EtOAc 8:2. The title compound was obtained as a white solid, yield 47%; m.p. 88–90 °C. ^1^H-NMR (400 MHz, CDCl_3_) *δ* (ppm): 2.39–2.52 (m, 8H, piperazine), 2.60 (t, 2H, J = 6.7 Hz, phthalimide-CH_2_CH_2_N), 3.44 (s, 2H, NCH_2_Ph), 3.78 (t, 2H, J = 6.7 Hz, phtalimide–CH_2_CH_2_N), 7.19–7.27 (m, 5H, aromatics, Ph–CH_2_), 7.67–7.69 and 7.80–7.82 (m, 4H, phtalimide). ESI-MS *m*/*z*: (IP: positive) 350 [M + H]^+^ [13].

##### 2-(2-(4-(2-Chlorobenzyl)piperazin-1-yl)ethyl)isoindoline-1,3-dione (**8b**)

Starting from 2-chlorobenzyl bromide, eluent: 100% EtOAc. The title compound was obtained as a yellow solid, yield 31%. ^1^H-NMR (300 MHz, CDCl_3_) *δ* (ppm): 2.37–2.74 (m, 10H, 8H piperazine + 2H phthalimide-CH_2_CH_2_N), 3.58 (s, 2H, NCH_2_Ph), 3.82 (t, 2H, J = 6.7 Hz, phtalimide–CH_2_CH_2_N), 7.12–7.23 and 7.29–7.49 (m, 4H, Ph–CH_2_), 7.64–7.73 and 7.80–7.92 (m, 4H, phtalimide). GS-MS *m*/*z* (%): 385 (1) [M + 2]^+^, 383 (4) [M]^+^, 223 (100), 125 (38).

##### 2-(2-(4-(2-Methylbenzyl)piperazin-1-yl)ethyl)isoindoline-1,3-dione (**8c**)

Starting from 2-methylbenzyl bromide, eluent: 100% EtOAc. The title compound was obtained as a white solid, yield 54%. ^1^H-NMR (300 MHz, CDCl_3_) *δ* (ppm): 2.34 (s, 3H, CH_3_), 2.41–2.65 (m, 10H, 8H piperazine + 2H, phthalimide-CH_2_CH_2_N), 3.41 (s, 2H, NCH_2_Ph), 3.81 (t, 2H, J = 6.5 Hz, phtalimide–CH_2_CH_2_N), 7.11–7.15 and 7.21–7.26 (m, 4H, aromatics), 7.68–7.73 and 7.83–7.86 (m, 4H, phtalimide); ^13^C-NMR (125 MHz, CDCl_3_) *δ* (ppm): 19.2, 35.4, 55.7, 60.7, 123.2, 125,4, 126.9, 129.8, 130.1, 132.2, 133.8, 136.5, 137.5, 168.3. GS-MS *m*/*z* (%): 363 (6) [M]^+^, 203 (100).

##### 2-(2-(4-(2-Fluorobenzyl)piperazin-1-yl)ethyl)isoindoline-1,3-dione (**8d**)

Starting from 2-fluorobenzyl bromide. The title compound was obtained as a yellow solid, yield 40%. ^1^H-NMR (300 MHz, CDCl_3_) *δ* (ppm): 2.56–2.62 (m, 10H, 8H piperazine + 2H, phthalimide-CH_2_CH_2_N), 3.56 (s, 2H, NCH_2_Ph), 3.80 (t, 2H, J = 6.4 Hz, phtalimide–CH_2_CH_2_N), 6.98–7.06 and 7.23–7.36 (m, 4H, Ph–CH_2_), 7.69–7.73 and 7.81–7.84 (m, 4H, phtalimide). GS-MS *m*/*z* (%): 367 (5) [M]^+^, 207 (100), 109 (41).

##### 2-(2-(4-(2-Methoxybenzyl)piperazin-1-yl)ethyl)isoindoline-1,3-dione (**8e**)

Starting from 3-methoxybenzyl bromide, eluent: 100% EtOAc. The title compound was obtained as a white solid, yield 30%. ^1^H-NMR (300 MHz, CDCl_3_) *δ* (ppm): 2.40–2.70 (m, 10H, 8H piperazine + 2H, phthalimide-CH_2_CH_2_N), 3.44–3.83 (m, 7H, 2H NCH_2_Ph + 2H phtalimide–CH_2_CH_2_N + 3H OCH_3_), 6.75–6.88 and 7.17–7.26 (m, 4H, Ph–CH_2_), 7.67–7.73 and 7.80–7.85 (m, 4H, phtalimide); ^13^C-NMR (125 MHz, CDCl_3_) *δ* (ppm): 35.3, 53.0, 55.2, 55.7, 62.9, 112.5, 114.6, 121.5, 123.2, 129.1, 132.2, 133.8, 159.6, 168.3. GS-MS *m*/*z* (%): 379 (9) [M]^+^, 219 (100).

#### 2.1.6. General Procedure for the Preparation of Free Amines **9a–e** and **10a–e**

The appropriate protected benzylated amine **7a–e** or **8a–e** (1.2 mmol) was mixed with MeNH_2_ 40% (*w*/*w*, aqueous solution, 10 mL) and left at RT for 72 h. Then, NaOH 20% (*w*/*w*, aq, 17 mL) was added to the solution and the resulting mixture was further stirred for 2 h. Finally, after the addition of sodium chloride (21.6 mmol), the aqueous portion was extracted with CH_2_Cl_2_ (three times). The collected organic layers were washed with brine, dried over anhydrous Na_2_SO_4_, filtered and concentrated under reduced pressure, affording the title compounds.

##### (1-Benzylpiperidin-4-yl)methanamine (**9a**)

Starting from 2-((1-benzylpiperidin-4-yl)methyl)isoindoline-1,3-dione (**7a**). The title compound was obtained as a yellow oil, yield 91%. ^1^H-NMR (300 MHz, DMSO-d_6_) *δ* (ppm): 1.00–1.23, 1.82–1.89 and 2.37–2.39 (m, 9H, piperidine), 2.77 (d, 2H, J = 11.5 Hz, NCH_2_CH), 3.41 (s, 2H, NCH_2_Ph), 7.20–7.33 (m, 5H, aromatics, Ph–CH_2_). ESI-MS *m*/*z*: (IP: positive) 205 [M + H]^+^ [13].

##### (1-(2-Chlorobenzyl)piperidin-4-yl)methanamine (**9b**)

Starting from 2-((1-(2-chlorobenzyl)piperidin-4-yl)methyl)isoindoline-1,3-dione (**7b**). The title compound was obtained as a pale-yellow oil, yield 78%. ^1^H-NMR (500 MHz, CDCl_3_) *δ* (ppm): 1.19–1.31 (m, 5H), 1.63–1.75 (m, 2H), 2.05 (app t, J = 11.2 Hz, 2H), 2.56 (d, J = 5.8 Hz, 2H), 2.86–2.96 (m, 2H), 3.58 (s, 2H), 7.14 (td, J = 7.6, 1.8 Hz, 1H), 7.20 (td, J = 7.4, 1.3 Hz, 1H), 7.31 (dd, J = 7.9, 1.4 Hz, 1H), 7.46 (dd, J = 1.7, 7.7 Hz, 1H) [27]. GS-MS *m*/*z* (%): 238 (3) [M]^+^, 125 (100).

##### (1-(2-Methylbenzyl)piperidin-4-yl)methanamine (**9c**)

Starting from 2-((1-(2-methylbenzyl)piperidin-4-yl)methyl)isoindoline-1,3-dione (**7c**). The title compound was obtained as a pale-yellow oil, yield 96%. ^1^H-NMR (300 MHz, CDCl_3_) *δ* (ppm): 1.07–1.46 (m, 3H), 1.56–1.75 (m, 4H), 1.85–2.06 (m, 2H), 2.35 (s, 3H), 2.57 (d, J = 6.0 Hz, 2H), 2.79–2.96 (m, 2H), 3.42 (s, 2H), 7.35–7.05 (m, 4H) [27]. GS-MS *m*/*z* (%): 218 (6) [M]^+^, 96 (100).

##### (1-(2-Fluorobenzyl)piperidin-4-yl)methanamine (**9d**)

Starting from 2-((1-(2-fluorobenzyl)piperidin-4-yl)methyl)isoindoline-1,3-dione (**7d**). The title compound was obtained as a pale-yellow oil, yield 98%. ^1^H-NMR (300 MHz, CDCl_3_) *δ* (ppm): 1.18–1.30, 1.62–1.76, 1.93–2.07 (m, 7H, piperidine), 2.53 (d, J = 5.9 Hz, 2H, NCH_2_CH), 2.86–2.93 (m, 2H, piperidine), 3.54 (s, 2H, s, 2H, NCH_2_Ph), 6.92–7.11 and 7.13–7.39 (m, 4H, aromatics); ^13^C NMR (75 MHz, CDCl_3_) *δ* (ppm): 29.3, 35.8, 45.5, 53.0, 55.4, 115.1, 123.8, 126.3, 128.8, 131.7, 160.4. GS-MS *m*/*z* (%): 222 (7) [M]^+^, 109 (100).

##### (1-(3-Methoxybenzyl)piperidin-4-yl)methanamine (**9e**)

Starting from 2-((1-(3-methoxybenzyl)piperidin-4-yl)methyl)isoindoline-1,3-dione (**7e**). The title compound was obtained as a yellow oil, yield 83%. ^1^H-NMR (500 MHz, CDCl_3_) δ (ppm): 1.16–1.33, 1.64–1.72, 1.88–1.99 (m, 7H, piperidine), 2.56 (d, J = 6.2 Hz, 2H, NCH_2_CH), 2.87–2.92 (m, 2H, piperidine), 3.46 (s, 2H, NCH_2_Ph), 3.81 (s, 3H, OCH_3_), 6.77–6.80, 6.87–6.91 and 7.18–7.24 (m, 4H, aromatics). GS-MS *m*/*z* (%): 234 (9) [M]^+^, 121 (80), 96 (100).

##### 2-(4-Benzyl-1-piperazine-1-yl)ethanamine (**10a**)

Starting from 2-(2-(4-benzylpiperazin-1-yl)ethyl)isoindoline-1,3-dione (**8a**). The title compound was obtained as a yellow oil, yield 99%. ^1^H NMR (400 MHz, CDCl_3_) *δ* (ppm): 2.35–2.43 (m, 10H, 8H piperazine + 2H NH_2_CH_2_CH_2_N), 2.73 (t, 2H, J = 6.1 Hz, NH_2_CH_2_CH_2_N), 3.46 (s, 2H, NCH_2_Ph), 7.19–7.27 (m, 5H, aromatics, Ph–CH_2_). ESI-MS *m*/*z*: (IP: positive) 220 [M + H]^+^ [13].

##### 2-(4-(2-Chlorobenzyl)piperazine-1-yl)ethanamine (**10b**)

Starting from 2-(2-(4-(2-chlorobenzyl)piperazin-1-yl)ethyl)isoindoline-1,3-dione (**8b**). The title compound was obtained as a yellow oil, yield 99%. ^1^H-NMR (500 MHz, DMSO-*d_6_*), *δ* (ppm): 2.24 (t, 2H, J = 6.5 Hz, NH_2_CH_2_CH_2_N), 2.28–2.44 (m, 8H piperazine), 2.45–2.49 (m, 2H, NH_2_), 2.56 (t, 2H, J = 6.5 Hz, NH_2_CH_2_CH_2_N), 3.51 (s, 2H, NCH_2_Ph), 7.22–7.31, 7.37–7.40 and 7.42–7.46 (m, 4H, aromatics). GS-MS *m*/*z* (%): 255 (1) [M + 2]^+^, 253 (1) [M]^+^, 223 (100), 180 (12), 125 (71).

##### 2-(4-(2-Methylbenzyl)piperazine-1-yl)ethanamine (**10c**)

Starting from 2-(2-(4-(2-methylbenzyl)piperazin-1-yl)ethyl)isoindoline-1,3-dione (**8c**). The title compound was obtained as a pale-yellow oil, yield 97%. ^1^H-NMR (300 MHz, CDCl_3_), *δ* (ppm): 1.90 (s, 3H, CH_3_), 2.34–2.45 (m, 10H, 8H piperazine + 2H NH_2_CH_2_CH_2_N), 2.77 (t, 2H, J = 6.2 Hz, NH_2_CH_2_CH_2_N), 3.44 (s, 2H, NCH_2_Ph), 7.12–7.15 and 7.23–7.25 (m, 4H, aromatics); ^13^C-NMR (75 MHz, CDCl_3_) *δ* (ppm): 19.2, 38.7, 53.2, 53.4, 60.7, 61.0, 125.4, 127.0, 129.8, 130.2, 136,4, 137.5. GS-MS *m*/*z* (%): 233 (1) [M]^+^, 203 (100).

##### 2-(4-(2-Fluorobenzyl)piperazine-1-yl)ethanamine (**10d**)

Starting from 2-(2-(4-(2-fluorobenzyl)piperazin-1-yl)ethyl)isoindoline-1,3-dione (**8d**). The title compound was obtained as a yellow oil, yield 82%. ^1^H-NMR (300 MHz, CDCl_3_), *δ* (ppm): 2.36–2.43 (m, 10H, 8H piperazine + 2H NH_2_CH_2_CH_2_N), 2.77 (t, 2H, J = 6.4 Hz, NH_2_CH_2_CH_2_N), 3.59 (s, 2H, NCH_2_Ph), 6.99–7.11, 7.19–7.26 and 7.30–7.38 (m, 4H, aromatics). GS-MS *m*/*z* (%): 237 (1) [M]^+^, 207 (100), 164 (16), 109 (85).

##### 2-(4-(3-Methoxylbenzyl)piperazine-1-yl)ethanamine (**10e**)

Starting from 2-(2-(4-(3-methoxylbenzyl)piperazin-1-yl)ethyl)isoindoline-1,3-dione (**8e**). The title compound was obtained as a pale-yellow oil, yield 92%. ^1^H-NMR (300 MHz, CDCl_3_) *δ* (ppm): 1.72 (s, 2H), 2.42 (t, J = 6 Hz, 2H), 2.49 (bs, 8H), 2.80 (t, J = 6 Hz, 2H), 3.48 (s, 2H), 3.81 (s, 3H), 6.82–6.76 (m, 1H), 6.93–6.88 (m, 2H), 7.23 (t, J = 8 Hz, 1H); other spectroscopic data agree with the literature [28].

#### 2.1.7. General Procedure for the Preparation of the Final Amides **11a–e**, **12a–e** and **13**

To a solution of **3** or **4** (0.315 mmol, 1 eq) in anhydrous DMF (2 mL) cooled in an ice bath and under inert atmosphere, *N*,*N*-diisopropylethylamine (DIEA, 0.95 mmol, 3 eq) was added and the mixture was stirred at 0 °C for 10 min. Then, 2-(1*H*-benzotriazole-1-yl)-1,1,3,3-tetramethylaminium tetrafluoroborate (TBTU, 0.47 mmol, 1.5 eq) and further 2 mL of anhydrous DMF were added and the reaction was stirred for 20 min at 0 °C and for 2 h at RT. At the end, a solution of the suitable amine **9a–e** or **10a–e** (0.47, 1.5 eq) in anhydrous DMF (3 mL) was added dropwise. The reaction was stirred under inert atmosphere at room temperature for 24 h. Then, the solvent was removed *in vacuo* and the resultant residue was treated with ethyl acetate or dichloromethane and washed with 2 N NaOH solution (three times) and brine (two times). The organic portion was dried over anhydrous Na_2_SO_4_, filtered and concentrated to dryness. The so obtained crude products were purified through column chromatography in various conditions.

##### *N*-((1-Benzylpiperidin-4-yl)methyl)-10*H*-phenothiazine-2-carboxamide (**11a**)

Starting from (1-benzylpiperidin-4-yl)methanamine (**9a**) and 10*H*-phenothiazine-2-carboxylic acid (**4**), eluent: CHCl_3_/MeOH 95:5. The title compound was obtained as a pale-yellow solid, yield 86%; m.p. = 169–170 °C. ^1^H-NMR (300 MHz, CDCl_3_), *δ* (ppm): 1.03–1.24, 1.54–1.67, 1.80–1.90, 2.68–2.85 and 3.27–3.33 (m, 9H, piperidine), 3.07 (t, 2H, J = 6.0 Hz, NHCH_2_CH), 3.43 (s, 2H, NCH_2_Ph), 6.59–6.77, 6.82–6.99, 7.03–7.35, 8.25–8.37 and 8.63–8.72 (m, 13H, 12H aromatics + 1H NH); ^13^C-NMR (75 MHz, CDCl_3_), δ (ppm): 30.1, 36.0, 45.1, 53.3, 62.7, 113.8, 115.0, 116.1, 120.4, 120.5, 122.4, 126.1, 126.7, 127.3, 128.2, 129.2, 134.7, 142.0, 142.3, 166.26. HRMS (C_26_H_27_N_3_OS-H^-^): calculated: 428.1802; found: 428.1786.

##### *N*-((1-(2-Chlorobenzyl)piperidin-4-yl)methyl)-10*H*-phenothiazine-2-carboxamide (**11b**)

Starting from (1-(2-chlorobenzyl)piperidin-4-yl)methanamine (**9b**) and 10*H*-phenothiazine-2-carboxylic acid (**4**), eluent: CHCl_3_/MeOH 95:5. The title compound was obtained as a brown solid, yield 40%; m.p. = 152–155 °C. ^1^H-NMR (300 MHz, CDCl_3_), *δ* (ppm): 1.23–1.40, 1.52–1.75, 2.00–2.14, 2.84–3.01 (m, 9H, piperidine), 3.33 (t, 2H, J = 5.2 Hz, NHCH_2_CH), 3.59 (s, 2H, NCH_2_Ph), 6.08–6.17, 6.52–6.61, 6.78–7.36 and 7.41–7.50 (m, 12H, 11H aromatics + 1H NH); ^13^C-NMR (126 MHz, CD_3_OD), δ (ppm): 28.5, 35.1, 44.5, 52.7, 58.0, 112.6, 114.1, 116.4, 119.7, 121.8, 122.1, 125.6, 125.9, 126.8, 127.3, 129.3, 131.8, 133.6, 134.7, 141.7, 142.6, 168.6. HRMS (C_26_H_26_ClN_3_OS-H^-^): calculated: 462.1412; found: 462.1395.

##### *N*-((1-(2-Methylbenzyl)piperidin-4-yl)methyl)-10*H*-phenothiazine-2-carboxamide (**11c**)

Starting from (1-(2-methylbenzyl)piperidin-4-yl)methanamine (**9c**) and 10*H*-phenothiazine-2-carboxylic acid (**4**), eluent: CHCl_3_/MeOH 95:5. The title compound was obtained as a pale-yellow solid, yield 21%; m.p. = 165–168 °C. 1H-NMR (300 MHz, CDCl_3_), *δ* (ppm): 1.20–1.40, 1.59–1.73, 1.91–2.06 and 2.85–2.93 (m, 9H, piperidine), 2.34 (s, 3H, CH_3_), 3.29 (t, J = 6.0 Hz, 2H, NHCH_2_CH), 3.44 (s, 2H, NCH_2_Ph), 6.22–6.32 (m, 1H, NH), 6.53–6.63, 6.74–7.30 (m, 11H, aromatics); ^13^C-NMR (75 MHz, CDCl_3_), *δ* (ppm): 19.3, 29.7, 35.8, 45.3, 53.2, 60.4, 113.3, 114.7, 117.2, 119.9, 122.7, 125.6, 125.7, 126.3, 126.6, 127.3, 127.6, 130.0, 130.3, 133.8, 137.4, 141.1, 142.1, 167.0. HRMS (C_27_H_29_N_3_OS-H^-^): calculated: 442.1959; found: 442.1953.

##### *N*-((1-(2-Fluorobenzyl)piperidin-4-yl)methyl)-10*H*-phenothiazine-2-carboxamide (**11d**)

Starting from (1-(2-fluorobenzyl)piperidin-4-yl)methanamine (**9d**) and 10*H*-phenothiazine-2-carboxylic acid (**4**), eluent: CHCl_3_/MeOH 95:5. The title compound was obtained as a yellow solid, yield 20%; m.p. = 168–170 °C. ^1^H-NMR (300 MHz, CDCl_3_), *δ* (ppm): 1.31–1.41, 1.54–1.70, 1.99 –2.07, 2.65–2.95, (m, 9H, piperidine), 3.26 (t, J = 6.2 Hz, 2H, NHCH_2_CH), 3.59 (s, 2H, NCH_2_Ph), 6.45 (s, 1H, NH), 6.56–6.62, 6.75–6.96, 6.99–7.14, 7.20–7.26 and 7.34–7.40 (m, 11H, aromatics); ^13^C-NMR (126 MHz, CDCl_3_), *δ* (ppm): 29.8, 35.8, 45.5, 53.0, 55.4, 113.4, 114.7, 115.2 (d, J2 = 22 Hz), 117.0, 119.7, 122.5, 122.6, 123.8, 126.4 (d, J2 = 40 Hz), 127.6, 128.8 (d, J3 = 7.6 Hz), 131.7 (d, J4 = 3.8 Hz), 133.7, 141.2, 142.2, 161.4 (d, J1 = 246 Hz), 167.2. HRMS (C_26_H_26_FN_3_OS-H^-^): calculated: 446.1708; found: 446.1693.

##### *N*-((1-(3-Methoxybenzyl)piperidin-4-yl)methyl)-10*H*-phenothiazine-2-carboxamide (**11e**)

Starting from (1-(3-methoxybenzyl)piperidin-4-yl)methanamine (**9e**) and 10*H*-phenothiazine-2-carboxylic acid (**4**), eluent: CH_2_Cl_2_/MeOH 8:2. The title compound was obtained as a pink solid, yield 21%; m.p. = 148–150 °C. ^1^H-NMR (500 MHz, CDCl_3_), *δ* (ppm): 1.29–1.42, 1.56–1.75, 1.91–2.02, 2.87–2.94 (m, 9H, piperidine), 3.32 (t, 2H, J = 6.0 Hz, NHCH_2_CH), 3.47 (s, 2H, NCH_2_Ph), 3.81 (s, 3H, OCH_3_), 6.08–6.18, 6.52–6.57, 6.75–7.08 and 7.19–7.25 (m, 12H, 11H aromatics + 1H NH). ^13^C-NMR (126 MHz, DMSO-d_6_), *δ* (ppm): 34.9, 40.8, 49.9, 58.1, 60.1, 67.5, 117.4, 118.5, 119.3, 119.7, 120.9, 125.1, 125.3, 126.1, 127.2, 130.9, 131.4, 132.9, 134.3, 139.4, 145.3, 146.7, 147.1, 164.4, 171.1. HRMS (C_27_H_29_N_3_O_2_S + Na^+^): calculated: 482.1873; found: 485.1887.

##### *N*-(2-(4-Benzylpiperazin-1-yl)ethyl)-10*H*-phenothiazine-2-carboxamide (**12a**)

Starting from 2-(4-benzyl-1-piperazine-1-yl)ethanamine (**10a**) and 10*H*-phenothiazine-2-carboxylic acid (**4**) eluent: CH_2_Cl_2_/MeOH 95:5. The title compound was obtained as a pale-yellow solid, yield 21%; m.p. = 190–194 °C. ^1^H-NMR (500 MHz, CDCl_3_), *δ* (ppm): 2.53–2.61 (m, 10H, m, 10H, 8H piperazine + 2H NHCH_2_CH_2_N), 3.50 (t, 2H, J = 5.9 Hz, NHCH_2_CH_2_N), 3.52 (s, 2H, NCH_2_Ph), 6.15 (s, 1H, NH), 6.55–6.57, 6.80–6.84, 6.94–7.01, 7.06–7.10 and 7.30–7.34 (m, 12H, aromatics). ^13^C-NMR (126 MHz, CDCl_3_), *δ* (ppm): 36.4, 52.8, 53.0, 56.2, 63.0, 113.6, 114.7, 117.2, 119.8, 122.5, 122.6, 126.3, 126.6, 127.1, 127.6, 128.2, 129.2, 133.6, 137.9, 141.2, 142.1, 166.7. HRMS (C_26_H_28_N_4_OS + H^+^): calculated: 445.2057; found: 445.2063.

##### *N*-(2-(4-(2-Chlorobenzyl)piperazin-1-yl)ethyl)-10*H*-phenothiazine-2-carboxamide (**12b**)

Starting from 2-(4-(2-chlorobenzyl)piperazine-1-yl)ethanamine (**10b**) and 10*H*-phenothiazine-2-carboxylic acid (**4**), eluent: CH_2_Cl_2_/MeOH 9:1. The title compound was obtained as a yellow solid, yield 83%; m.p. = 148–150 °C. ^1^H-NMR (300 MHz, CDCl_3_), *δ* (ppm): 2.43–2.66 (m, 10H, 8H piperazine + 2H NHCH_2_CH_2_N), 3.41–3.55 (m, 2H, NHCH_2_CH_2_N), 3.64 (s, 2H, NCH_2_Ph), 6.13 (s, 1H, NH), 6.51–6.60, 6.75–6.87 6.89–7.12, 7.30–7.38 and 7.42–7.48 (m, 11H, aromatics). ^13^C-NMR (126 MHz, CDCl_3_), *δ* (ppm): 36.2, 52.7, 52.9, 56.4, 59.1, 113.5, 114.7, 117.1, 119.9, 122.6, 122.6, 126.3, 126.5, 126.6, 127.6, 128.2, 129.5, 130.7, 133.5, 134.3, 135.6, 141.2, 142.1, 166.8. HRMS (C_26_H_27_ClN_4_OS + H^+^): calculated: 479.1667; found: 479.1673.

##### *N*-(2-(4-(2-Methylbenzyl)piperazin-1-yl)ethyl)-10*H*-phenothiazine-2-carboxamide (**12c**)

Starting from 2-(4-(2-methylbenzyl)piperazine-1-yl)ethanamine (**10c**) and 10*H*-phenothiazine-2-carboxylic acid (**4**), eluent: CHCl_3_/MeOH 95:5. The title compound was obtained as a light brown solid, yield 53%; m.p. = 145–147 °C. ^1^H-NMR (300 MHz, CDCl_3_), *δ* (ppm): 2.35 (s, 3H, CH_3_), 2.53–2.68 (m, 10H, 8H piperazine + 2H NHCH_2_CH_2_N), 3.74 (s, 2H, CH_2_, NCH_2_Ph), 3.51–3.61 (m, 2H, CH_2_, NHCH_2_CH_2_N), 6.41 (s, 1H, NH), 6.57–6.60 and 6.79–7.22 (m, 11H, aromatics); ^13^C NMR (75 MHz, CDCl_3_) *δ* (ppm): 19.2, 36.0, 52.3, 53.0, 56.6, 60.5, 112.5, 120.2, 122.6, 125.5, 126.3, 126.6, 127.2, 127.6, 129.8, 130.3, 135.9, 137.5, 141.1, 166.7. HRMS (C_27_H_30_N_4_OS + H^+^): calculated: 459.2213; found: 459.2222.

##### *N*-(2-(4-(2-Fluorobenzyl)piperazin-1-yl)ethyl)-10*H*-phenothiazine-2-carboxamide (**12d**)

Starting from 2-(4-(2-fluorobenzyl)piperazine-1-yl)ethanamine (**10d**) and 10*H*-phenothiazine-2-carboxylic acid (**4**), eluent: CH_2_Cl_2_/MeOH 95:5. The title compound was obtained as a pale-yellow solid, yield 13%; m.p. = 185–187 °C. ^1^H-NMR (300 MHz, CDCl_3_), *δ* (ppm): 2.57–2.65 (m, 10H, m, 10H, 8H piperazine + 2H NHCH_2_CH_2_N), 3.47–3.51 (m, 2H, NHCH_2_CH_2_N), 3.61 (s, 2H, NCH_2_Ph), 6.16 (s, 1H, NH), 6.56–6.59, 6.82–7.00, 7.03–7.13, 7.21–7.23 and 7.34–7.39 (m, 11H, aromatics). ^13^C-NMR (126 MHz, CDCl_3_), *δ* (ppm): 36.1, 52.4, 52.8, 55.1, 56.3, 113.4, 114.6, 115.3 (d, J2 = 22.7 Hz), 117.3, 120.1, 122.6, 122.8, 123.9, 124.2, 124.4, 126.5 (d, J2 = 37 Hz), 127.6, 128.9 (d, J3 = 8.2 Hz), 131.5 (d, J4 = 5.0 Hz), 133.53, 141.0, 141.9, 161.4 (d, J1 = 247 Hz), 166.6. HRMS (C_26_H_27_N_4_OSF + Na^+^): calculated: 485.1782; found: 485.1783.

##### *N*-(2-(4-(2-Methoxybenzyl)piperazin-1-yl)ethyl)-10*H*-phenothiazine-2-carboxamide (**12e**)

Starting from 2-(4-(2-methoxybenzyl)piperazine-1-yl)ethanamine (**10e**) and 10*H*-phenothiazine-2-carboxylic acid (**4**), eluent: CHCl_3_/MeOH 95:5. The title compound was obtained as a yellow solid, yield 77%; m.p. = 166–167 °C. ^1^H-NMR (300 MHz, CDCl_3_), *δ* (ppm): 2.26–2.61 (m, 10H, 8H piperazine + 2H NHCH_2_CH_2_N), 3.49–3.53 (m, 4H, 2H NCH_2_Ph + 2H NHCH_2_CH_2_N), 3.80 (s, 3H, OCH3), 6.50 (s, 1H, NH), 6.57–6.59, 6.79–6.82, 6.89–6.99, 7.05–7.07 and 7.15–7.24 (m, 11H, aromatics); ^13^C NMR (75 MHz, CDCl_3_) *δ* (ppm): 36.1, 52.4, 52.8, 55.2, 56.5, 62.6, 112.6, 113.4, 114.6, 117.1, 120.0, 121.4, 122.6, 126.3, 126.6, 127.6, 129.2, 133.4, 139.3, 141.1, 159.7, 166.8. HRMS (C_27_H_30_N_4_O_2_S + H^+^): calculated: 475.2162; found: 475.2171.

##### 10-Acetyl-*N*-(2-(4-(2-chlorobenzyl)piperazin-1-yl)ethyl)-10*H*-phenothiazine-2-carboxamide (**13**)

Starting from 2-(4-(2-chlorobenzyl)piperazine-1-yl)ethanamine (**10b**) and 10-acetyl-10*H*-phenothiazine-2-carboxylic acid (**3**), eluent: EtOAc/MeOH 97:3. The title compound was obtained as a yellow solid, yield 21%; m.p. = 98–100 °C. ^1^H-NMR (300 MHz, CDCl_3_), δ (ppm): 2.22 (s, 3H, COCH_3_), 2.48–2.68 (m, 10H, 8H piperazine + 2H NHCH_2_CH_2_N), 3.50–3.58 (m, 2H, NHCH_2_CH_2_N), 3.64 (s, 2H, NCH_2_Ph), 6.86–7.00 (m, 1H, NH), 7.14–7.38, 7.40–7.54, 7.57–7.66 and 7.92–7.97 (m, 11H, aromatics). ^13^C-NMR (126 MHz, CDCl_3_), *δ* (ppm): 23.0. 36.4, 52.9, 53.0, 56.3, 59.1, 125.2, 125.9, 126.6, 127.1, 127.3, 127.8, 128.0, 128.2, 129.4, 130.7, 133.7, 134.3, 135.7, 138.5, 167.2, 169.3. HRMS (C_28_H_29_ClN_4_O_2_S + Na^+^): calculated: 543.1592; found: 543.1607.

### 2.2. Biological Methods

#### 2.2.1. AChE and BChE Inhibition

The experiments were carried out using a properly modified version of Ellman’s spectrophotometric assay [29], adapted to a 96-well plate procedure [30]. All reagents, including enzymes, were purchased from common suppliers. Incubation of the proper enzyme with the single compound at different concentrations was performed in clear flat-bottomed, 96-well plates (Greiner Bio-One GmbH, Frickenhausen, Germany) and in duplicate. When necessary, IC_50_ was determined by using seven different solutions of the inhibitor and prepared by diluting a stock solution 1000 μM (DMSO) with the work buffer in an opportune concentration range (generally from 10^−4^ to 10^−10^ M). Fluorescence measures were carried out using Tecan Infinite M1000 Pro multiplate reader (Tecan, Cernusco S.N., Italy). IC_50_ values and inhibition values were calculated as the mean of three independent experiments and are expressed as mean ± SEM [13].

#### 2.2.2. Inhibition of Aβ_40_ Aggregation

ThT fluorescence in the presence of Aβ was measured for each compound according a previously described procedure [30]. The samples were co-incubated and mixed in 96-well black, non-binding microplates (Greiner Bio-One GmbH, Frickenhausen, Germany), incubating Aβ_40_ (EZBiolab, Carmel, IN, USA) at a final concentration of 30 µM with each inhibitors at a final concentration of 10 µM in PBS (pH 7.4) containing 2% 1,1,1,3,3,3-hexafluoro-2-propanol (HFIP). After 2 h at 25 °C, a 25 µM ThT solution was added and fluorescence was determined using the multi-plate reader Tecan Infinite M1000 Pro (Tecan, Cernusco S.N., Italy). The results are reported as mean of three independent experiments ± SEM [13].

#### 2.2.3. FAAH Inhibition

The fatty acid amide hydrolase inhibition was measured for the final compounds using 96-well black flat-bottom microtiter NBS plates (COSTAR flat black). The total volume was 150 µL: the single hybrid was pre-incubated at different concentrations with the enzyme (FAAH Human recombinant, Cayman Chemical, Ann Arbor, MI, USA) in the specific fluorometric assay buffer (tris-HCl 125 mM, Na_2_EDTA · 2H_2_O 1 mM, pH = 9.0) for ten minutes at RT, using an orbital shaker. A 50 μL solution of the substrate (7-amino-4-methyl-2H-1-benzopyran-2-one-5*Z*,8*Z*,11*Z*,14*Z*-eicosatetraen-amide, at a final concentration of 5 µM) was then added. The plates were then incubated for 1 h at 37 °C in a Tecan Infinite M1000 Pro (Tecan, Cernusco S.N., Italy), reading fluorescence values from each well every 30s (λ_ex_ = 340 nm, λ_em_ = 450 nm). The inhibitor activity was expressed as relative fluorescence units (RFU), using the values obtained from control wells lacking the inhibitor and blank wells lacking both inhibitor and enzyme to calculate percent inhibition values for each well. IC_50_ values are expressed as mean ± SEM of at least two independent measurements performed in triplicate [13].

#### 2.2.4. DPPH Assay

A previously reported method (Blois) with some modifications (Mishra, Ojha, & Chaudhury, 2012) was used to carry out the DPPH assay in 96-well microtiter plates. Each tested compound was dissolved in methanol, after which a freshly prepared solution of DPPH in methanol (100 µM final concentration) was added [31,32]. Following vigorous stirring, each mixture was left in the dark for 30 min at room temperature, after which absorbance values for each well were read at 520 nm using a spectrophotometric plate reader (VICTOR^3^ V multilabel plate reader, PerkinElmer). Antioxidant activity was determined as RSA% (radical scavenging activity). The value was calculated using following equation: RSA% = 100 × [(Ao − Ai)/Ao], where Ao and Ai represent DPPH absorbance in absence or in presence of antioxidant, respectively. Antiradical curves for calculating the EC_50_ values were obtained by testing each compound at several concentrations. Values of all parameters are expressed as mean ± SEM of at least three independent measurements in triplicate [31,32].

#### 2.2.5. Cultured Cells

Human hepatocellular liver carcinoma (HepG2) cell line and human neuroblastoma cell line, SH-SY5Y, were purchased from the American Type Culture Collection (ATCC, Manassas, VA, USA) and maintained at 37 °C in a humidified atmosphere (95% air and 5% carbon dioxide), and they were periodically screened for contamination. HepG2 cells were cultured in Eagle’s Minimum Essential Medium (MEM, Euroclone S.p.A., Pero, MI, Italy), supplemented with 10% Fetal Bovine Serum (FBS, Euroclone S.p.A., Pero, MI, Italy), 1% L-glutamine (Euroclone S.p.A., Pero, MI, Italy), 100 U/mL penicillin/streptomycin (Euroclone S.p.A., Pero, MI, Italy), 1% Non-Essential Amino Acids (NEAA, Euroclone S.p.A., Pero, MI, Italy). SH-SY5Y cells were grown in a 1:1 mixture of Dulbecco’s modified Eagle’s medium (DMEM)—high glucose (Euroclone S.p.A., Pero, MI, Italy) and Ham’s F12 Medium (Euroclone S.p.A., Pero, MI, Italy) supplemented with 10% FBS, 1% L-glutamine and 100 U/mL penicillin/streptomycin. For cell assays, cells were trypsinized using Trypsin-EDTA 1X in PBS (Euroclone S.p.A., Pero, MI, Italy).

#### 2.2.6. Dichlorofluorescein Assay

According to a slightly modified procedure already reported in the literature [33], the generation of ROS was determined using an oxidation-sensitive fluorescent probe, 2′,7′-dichlorodihydrofluorescein diacetate (DCFH-DA, D6665; Sigma-Aldrich, St. Louis, MO, USA). Briefly, viable cells were seeded in a black 96-well cell culture plate (Costar, Sigma-Aldrich, St. Louis, MO, USA) and after 24 h were incubated with different concentrations (0.1–100 µM) of each compound for 1 h at 37 °C in 5% CO_2_. DCFH-DA in medium without serum was added directly to each well at a final concentration of 25 μM, and the plate was incubated at 37 °C for 30 min at 37 °C in 5% CO_2_. After washing using PBS, 100 μM H_2_O_2_ in medium without serum was added to each well and the cells were incubated for an additional 30 min. The formation of fluorescent dichlorofluorescein (DCF) due to oxidation of DCFH in the presence of ROS, was read at 530 nm using a microplate reader Tecan Infinite M1000 Pro (Tecan, Cernusco S.N., Italy) and DMSO medium was used for control cells. The results are expressed as mean ± SEM of at least three independent measurements in triplicate.

#### 2.2.7. Cell Viability Assay

HepG2 and SH-SY5Y cell viability was assessed as previously reported by using a conventional 3-(4,5-dimethylthiazol-2-yl)-2,5-diphenyltetrazolium bromide (MTT) assay [3,34]. In brief, viable cells were seeded and grown for 24 h into a sterile 96-well cell culture cluster (Corning, Sigma-Aldrich, St. Louis, MO, USA) in a complete medium and maintained at 37 °C in a humidified atmosphere with 5% CO_2_. The cells were then added with different concentrations of each compound for a period of 1 h or 24 h. At the end, the culture medium was replaced by a solution of 0.5 mg/mL MTT (Sigma-Aldrich, St. Louis, MO, USA) in PBS. After 2 h of incubation at 37 °C in 5% CO_2_, the supernatant was carefully removed from each well, and the formazan crystals were dissolved in DMSO. The absorbance values were measured at 570 nm using Tecan Infinite M1000 Pro (Tecan, Cernusco S.N., Italy) and DMSO medium as blank solution. The results were reported as % cell viability = [optical density (OD) of tested compound/medium OD of control cells] × 100. The results are expressed as mean ± SEM of at least three independent measurements in triplicate.

### 2.3. Computational Methods

#### 2.3.1. Docking Studies

The complexes of AChE (chain A; PDB code 6O4W) [35], BChE (PDB code 7BGC) [36], and FAAH (PDB code 4DO3) with the proper inhibitor were selected as targets for docking [37]. The X-ray structures were prepared for dockings with the Protein Preparation Wizard interface of Maestro: the ligand and water molecules were removed, while hydrogen atoms were added and their positions were optimized [38,39,40,41]. The ionization states of acid and basic residues were assigned according to PROPKA prediction at pH 7.0. Electrostatic charges for proteins atoms were loaded according to AMBER UNITED force field [38]. The convertion into 3D structures was performed using the Maestro software package starting from the SMILES string of each ligand [39]. Subsequently, the appropriate ionization was assigned with fixpka complement of QUACPAC [40], and the molecular skeleton was minimized performing a 10,000 steps of Steepest Descent with Open Babel [41] using the Universal Force Field while the molcharge complement of QUACPAC [40] was used in order to achieve Marsili-Gasteiger charges for the inhibitors. A 0.375 Å spaced 85 × 85 × 85 Å^3^ cubic box, having the barycenter on the co-crystallized inhibitors poses, was considered on affinity maps calculations for each enzyme, and the binding site available space was screened throughout 1000 runs of Lamarckian Genetic Algorithm (LGA) implemented in AUTODOCK 4.2.6 [42] using the GPU-OpenCL algorithm version [43]. Water molecules’ contribution in the binding was ensured with the hydration force field parameters [44], and the population size and the number of energy evaluation figures were set to 300 and 10,000,000, respectively [13]. The rank of docking poses was obtained by ESP, a rule based on energy (E = free energy of binding, the energy difference between the selected pose and the relative global minimum and the ligand efficacy), similarity (S = similarity as scored by the Tanimoto_Combo coefficient according to the shape matching algorithm ROCS [45]), and population (*p* = cluster member population) [13].

#### 2.3.2. Prediction of Pharmacokinetic Properties

The structures of compounds **11a–e**, **12a–e** and **13** were first optimized through Maestro [39]. Then, the pharmacokinetic properties were predicted using the software QikProp v.2.5 [46]. The determined parameters were: clogP (lipophilic character), logBB (the capacity to cross the blood–brain barrier), Caco-2 permeability (velocity of intestinal absorption), activity in the CNS, and Lipinski’s rule of 5.

## 3. Results and Discussion

### 3.1. Chemistry

The synthetic route for hybrids **11–13** necessitated the previous preparation of the phenothiazine acid intermediates (**3** and **4**) and of the aminoalkyl-piperidine and -piperazine intermediates (**9** and **10**), which were finally condensed via amide bond formation to obtain the final products (**11**, **12** and **13**).

**Scheme 1:**
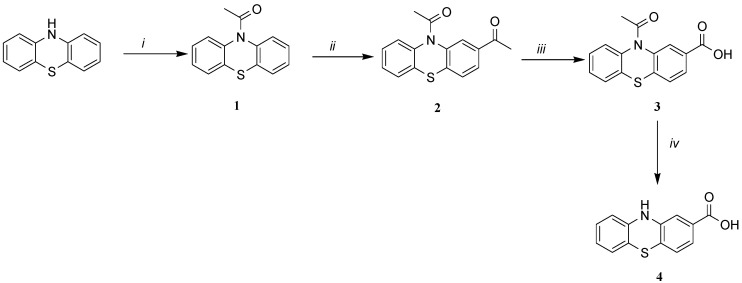
(**i**) Acetyl chloride, ZnCl_2_, CH_2_Cl_2_, 0 °C → r.t., 24 h; (**ii**) acetyl chloride, AlCl_3_, anhydrous CH_2_Cl_2_, 50 °C, 6 h → r.t., 15 h; (**iii**) sublimated I_2_, pyridine, 110 °C, 15 min → r.t., 20 h; (**iv**) 2N NaOH, 100 °C, 2 h.

Scheme 1 describes the synthesis of intermediates **3** and **4**. Commercial 10*H*-phenothiazine was acylated with acetyl chloride in the presence of ZnCl_2_, affording **1** [25]. Friedel–Crafts reaction with acetyl chloride led to **2**, that was treated with sublimated iodine, giving acid **3**. The subsequent basic hydrolysis allowed us to obtain acid **4** [47].

**Scheme 2:**
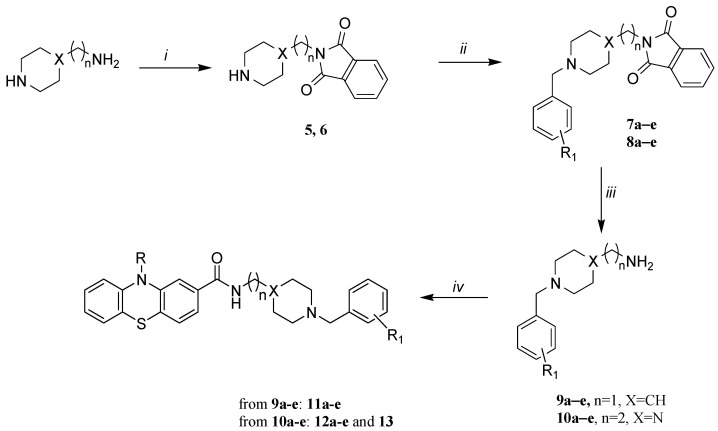
(**i**) Phthalic anhydride, 150 or 160 °C, 4 h; (**ii**) substituted benzyl bromide, triethylamine or KOH, 96° ethanol, r.t., 24 h; (**iii**) MeNH_2_ (40% *w*/*w*), r.t., 72 h; (**iv**) **3** or **4**, TBTU, DIEA, anhydrous DMF, 0 °C → r.t., overnight.

The synthesis of final compounds **11a–e**, **12a–e** and **13** is described in Scheme 2. The preparation of the aminoalkyl-piperidine and -piperazine intermediates (**9a–e** and **10a–e**) involves a selective protection of the primary alkylamine group of commercial 4-(aminomethyl)piperidine or 1-(2-aminoethyl)piperazine by a neat reaction with phthalic anhydride at 150–160 °C [48]. The subsequent *N*-benzylation of the cyclic amine involved a reaction with the suitable commercially available substituted benzyl bromide under basic conditions [18]. Deprotection of amino group with an aqueous solution of methylamine 40% (*w*/*w*) led to the free amines **9a–e** and **10a–e** [20]. Final compounds **11a–e**, **12a–e** and **13** were obtained by condensation of the primary amine group of **9a–e** or **10a–e** with the carboxylic group of **3** or **4** using 2-(1*H*-benzotriazole-1-yl)-1,1,3,3-tetramethylaminium tetrafluoroborate (TBTU) as coupling agent and *N*,*N*-diisopropylethylamine (DIEA) [49].

### 3.2. Biological Assays

The ability of the newly synthesized compounds **11a–e**, **12a–e**, and **13** to inhibit the enzymes AChE, BChE and FAAH and Aβ_1-40_ aggregation is reported in Table 1 as IC_50_ (μM) or as percentage of inhibition (I%) at a fixed ligand concentration (10 μM). Donepezil was used as reference compound for ChEs and Aβ, while JZL195 was used as reference compounds for FAAH.

#### 3.2.1. ChEs, FAAH, Aβ_1-40_ Aggregation Inhibition

The piperidine series (**11a–e**) shows moderate anti-AChE activity. In particular, the compounds with unsubstituted benzyl derivative (**11a**) and the one bearing the smallest fluorine *ortho*-substituent (**11d**) present the best activities, with IC_50_ values of 0.597 and 1.75 µM, respectively. **11a** shows selectivity towards AChE, while IC_50_ values of **11d** towards the two cholinesterases are comparable. Other compounds of the series, such as **11b**, **11c** and **11e**, are the most selective towards BChE.

The piperazine series (**12a–e** and **13**) shows moderate to good inhibition of AChE. In particular, the absence of substituents on the benzyl group (**12a**) or the presence of a halogen atom (**12b** and **12d**) in *ortho* position supports this activity. Acetylation of phenothiazine nitrogen atom in compound **13** results in lower activity against AChE, emphasizing the importance of the existence of a free nitrogen atom in the phenothiazine nucleus. This series exhibits a moderate activity against BChE, apart from compounds bearing a chlorine substituent in the *ortho* position on the benzyl group, such as **12b** and **13**, whose activity is poor (the percentage of inhibition is 15–27% at 10 µM). In particular, compounds **12c** and **12e** appear to be more selective towards BChE rather than AChE.

FAAH inhibition was measured via a fluorimetric enzyme assay, and the data are again reported in Table 1. Results obtained for the piperidine series (**11a–e**) are promising and within a relatively narrow range of IC_50_ (3.08–22.6 µM), probably because the proposed substituents are far from the pharmacophore. The IC_50_ values obtained for piperazines (**12a–e** and **13**) are also in the micromolar range (6.31–41.4 µM). It is noteworthy that the activity on this target is also affected by the functionalization of the phenothiazine nitrogen atom; in fact, the IC_50_ value of compound **13** is almost three-fold higher than that of the corresponding unsubstituted analogue **12b**. At any rate, the results shown in Table 1 evidence some beneficial effect on inhibitory capacity resulting from the presence of substituents when comparing to the non-substituted piperidine and piperazine hybrids (**11a** and **12a**).

Aβ_1-40_ in vitro aggregation was also evaluated. In the piperidine series, **11c** and **11d** are the most effective inhibitors on this target, with moderate activity, while for the piperazine series **12d** is the most active, presenting a good inhibition (I% at 10 µM = 60% ± 6); **12a** also shows a moderate inhibitory activity. As observed for cholinesterases, the acetylation of nitrogen atom in phenothiazine ring leads to a lack of activity of compound **13** at 10 µM. As reported in the past, the presence of a bulky heterocycle structure (in this case the phenothiazine, formerly the benzimidazole) seems to be confirmed as a critical structural requirement for this kind of activity [19].

These are the best results obtained so far for multi-target agents synthesized by our group: in a recent study on donepezil-like hybrids [13] we obtained compounds with very high activities on AChE (IC_50_ up to sub-nanomolar), moderate inhibitory abilities on FAAH (in the medium-micromolar range) and no activity on Aβ_1-40_ aggregation, except for a few derivatives at very high concentrations (100 µM). In other cases [15], the good activity on FAAH (low-micromolar range) was not associated with acceptable potencies against AChE and Aβ_1-40_ aggregation.

In another recent work of our group [18], the introduction of functional groups on the benzyl moiety able to increase or decrease the electron density led to a similar (2-fluorine) or worse (2-nitro or pyridine) AChE inhibition activity, whereas the activity on Aβ aggregation was only minimally affected. In the present study, however, SAR was extended to a higher number of substituents and biological targets, with results that can be finally discussed; in particular, for the piperazine series, 2-fluorine insertion on the benzyl ring of the piperazine series (**12d**) leads to a better activity on three targets out of four (namely AChE, Aβ_1-40_ and FAAH), compared to the unsubstituted hybrid (**12a**). The other substitutions lead to lower activities (more evident in **12e**) except for FAAH activity that is generally improved by the presence of any functional group. Similar observations were found for the piperidine series (**11a–e**), whose activity differs only for the three targets best affected (in this case BChE, Aβ_1-40_ and FAAH).

#### 3.2.2. Antioxidant Activity

The antioxidant activity of the hybrids **11a–e**, **12a–e**, and **13** was evaluated in vitro using the 2′,7′-dichlorodihydrofluorescein diacetate (DCFH-DA) cell-based assay. The test was based on measuring the reducing effect of the single compound against oxidation of 2′,7′-dichlorodihydrofluorescein (DCFH) to the fluorescent probe 2′,7′-dichlorofluorescein (DCF). Human hepatocellular carcinoma (HepG2) cells were chosen as the model considering their enhanced oxidative metabolism, which causes cellular oxidative stress and/or generates reactive metabolites. Thus, it may be assumed that HepG2 cells are suited to study protection against oxidative and cytotoxic effects, if any [50]. Furthermore, neuroblastoma SH-SY5Y cells have been used as a model for human neurons. Quercetin, a naturally occurring compound known to have strong antioxidant activity, has been used as positive control. The results are summarized in Table 2.

All phenothiazine/donepezil hybrids remarkably reduce H_2_O_2_-induced oxidation in both cell lines, with IC_50_ values ranging from 0.6 µM to 55.3 µM, and with most of the compounds being better antioxidants than quercetin. Among the piperidine analogues (**11a–e**), compound **11d**, bearing a 2-fluorine substituent on the benzyl moiety, showed the best antioxidant activity on HepG2 cell line with an IC_50_ value of 1.82 ± 0.60 µM, significantly lower than that of quercetin (12.5 ± 0.41 µM). A notable activity was also detected for compound **11e** (IC_50_ = 3.21 ± 0.50 µM) bearing a methoxy group at the *meta* position of the benzyl moiety. Though equally effective, a slightly lower activity was obtained with the unsubstituted analogue **11a** (IC_50_ = 8.31 ± 1.40 µM), followed by the 2-chloro and 2-methyl substituted analogues (**11b** and **11c**, respectively). Concerning the SH-SY5Y cell line, all piperidine derivatives performed as potent antioxidants with IC_50_ values ranging from 1.12 to 14.1 µM. Among piperazine derivatives (**12a–e**), the unsubstituted compound **12a** was the most active analogue (IC_50_ = 1.13 ± 0.41 µM) on HepG2 cells, whit the other derivatives showing the following trend **12c** > **12e** > **12d** > **12b**. Similarly, **12a** showed to have potent antioxidant activity on SH-SY5Y cells along with the 3-methoxybenzyl derivative **12e**, their IC_50_ values being 2.01 ± 0.61 µM and 1.93 ± 0.81 µM, respectively. Significant antioxidant activity was recorded for the other piperazine analogues **12b**, **12c**, and **12d** with IC_50_ values close to each other. It is interesting to note that among all the tested compounds, the only *N*-acetylated derivative (**13**) exhibited a noteworthy antioxidant capacity in both cellular models, being the best compound of the series on HepG2 cells with an IC_50_ value of 0.63 ± 0.10 µM.

All phenothiazine derivatives were subsequently evaluated for their reactivity with 1,1-diphenyl-2-picrylhydrazyl (DPPH), a violet-coloured stable radical that absorbs strongly at 517 nm, which allowed us to estimate their potential efficacy as scavengers of stable free radicals. Experiments were performed using gallic acid, a naturally occurring plant phenol with antioxidant activity, as the positive control. The EC_50_ values determined for each compound and gallic acid are summarized in Table 3.

Almost all compounds showed interesting reactivity with this radical, although to lesser extent than gallic acid, with EC_50_ values ranging from 0.082 to 0.231 μmol/μmol_DPPH_. The only exception to this behaviour was the *N*-acetyl derivative **13** that was not able to scavenge the DPPH radical with the same efficacy its (EC_50_ > 1 μmol/μmol_DPPH_). The best activity was achieved by phenothiazine derivative **11a** with an EC_50_ value amounting to 0.082 ± 0.007 μmol/μmol_DPPH_, close to that of the reference compound (0.059 μmol/μmol_DPPH_), although the introduction of the different substituents in both series had a similar effect (EC_50_ = 0.104–0.231 μmol/μmol_DPPH_). It is interesting to note that analogue **13**, which was completely inactive in the DPPH test, exhibited a noteworthy antioxidant capacity in both cellular models. This result may plausibly suggest that the presence of the NH residue is crucial for radical scavenging activity, eventually by formation of a resonance-stabilized free radical, which is more difficult if the nitrogen electron lone pair is involved in a N-CO amide bond. Thus, this leads us to hypothesize that compound **13** undergoes deacetylation at the cellular level which endows it with antioxidant activity. These results are consistent with those obtained in a recent work about phenothiazine-tacrine hybrids as potential anti-AD multi-target drugs [23]: the alternative design of this series could be a winning strategy to preserve this crucial activity. The present study represents a key step in our research: in the recent past we obtained hybrids with good multi-functional profile but moderate-low antioxidant activity [19], or interesting nature-inspired scaffolds [14] which did not maintain their antioxidant profiles once included in donepezil-like hybrid structures.

Furthermore, to rule out the potential cytotoxicity of the tested compounds, a MTT assay was performed on both cell lines at different times (1 h and 24 h) [51]. Tested compounds did not show any cytotoxicity at the incubation times used for the DCFH-DA assay (1 h). A slight decrease in viability was found at 24 h but with IC_50_ values clearly higher than the effective doses that were determined in the DCFDH-DA assay (Table 4).

#### 3.2.3. Multi-Target Profiles

**Figure 2:**
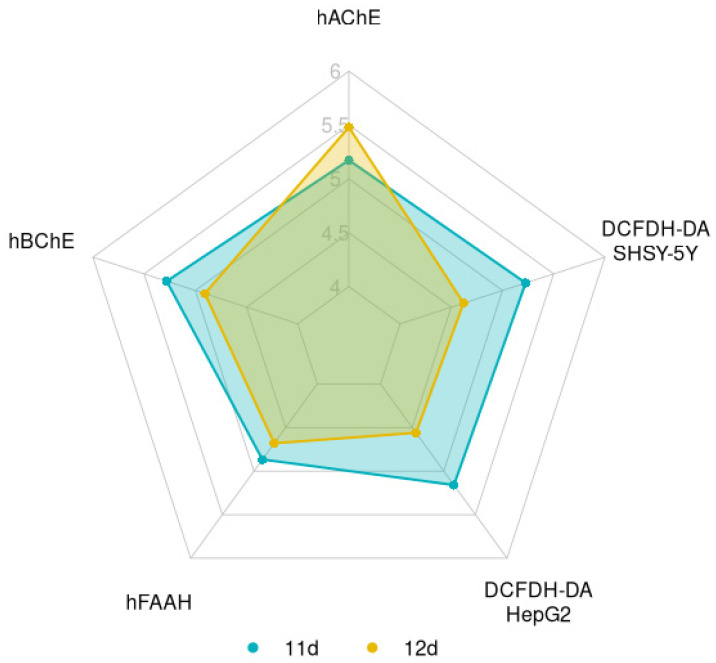
Summary chart representing the biological activities of **11d** (blue area) and **12d** (orange area). Data are reported as pIC_50_.

The representation of the main biological activities as pIC_50_ in a summary chart (Figure 2 and Figure S2) showed that the more promising compounds of the series were **11d** and **12d**, considering their interesting multi-target profiles.

### 3.3. Molecular Docking Calculations

The most versatile compound **11d** was used as a decoy to corroborate the biological activity data and the SAR of the novel phenothiazines via molecular docking to the binding site of the investigated enzymes. In the very first step of this in silico study, the molecular similarity of our ligand with the known AChE inhibitor donepezil and its complementarity with respect to the enzyme’s binding site was considered. The catalytic triad CT (Ser203, Glu334, His447), the catalytic anionic site CAS (Trp86 and Phe338) and the peripheral anion site PAS (Tyr72, Tyr124 and Trp286) are well-known as the critical anchoring spots on the binding sites of the enzyme, as well as the entry pathway where inhibitors might adopt *outward*–*inward* orientations. Thus, the hAChE/donepezil X-ray complex, PDB code: 6O4W [35] was then enrolled to dock **11d** whose relative binding poses were carefully filtered by the **ESP** rule with the following parameters: **FEB** < −10.00, **ΔE** < 2.00, **EFF** < −0.300, **SIM** > 0.800, **POP** > 20 (see methods).

**Figure 3:**
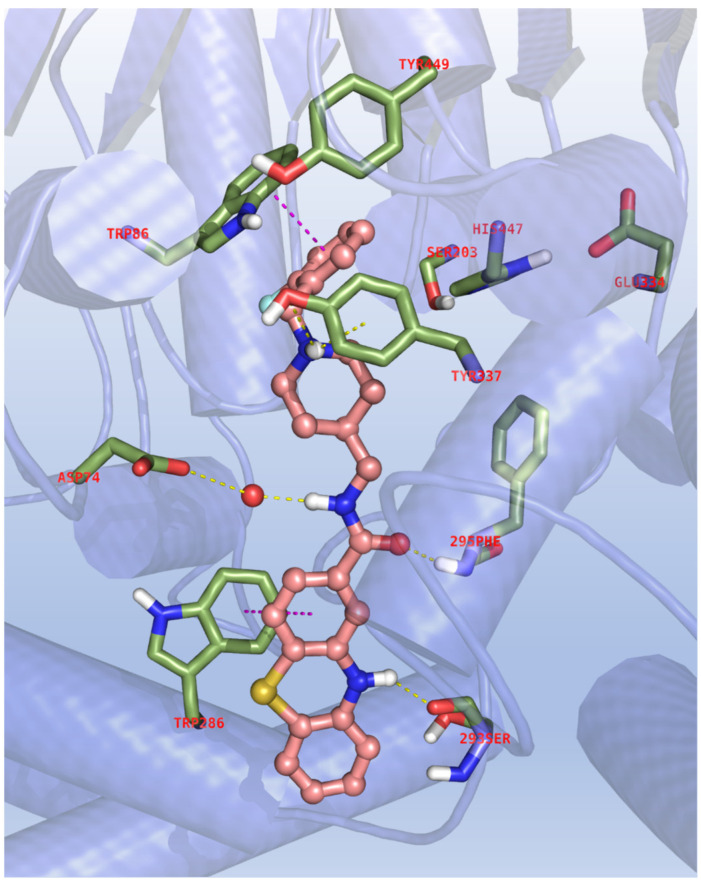
Binding mode for **11d** to the AChE active site. In the interaction pattern scheme, hydrogen bonds and π-π stackings are depicted in yellow and magenta, respectively.

As shown in Figure 3, **11d** largely seals all the available space at the base and at the opening of the AChE active center gorge, with the benzyl group and the protonated piperidine amino group producing, respectively, significant π-π stacking with the CAS Trp286 and a hydrogen bond, in combination with cation–π interactions, with the side chain of Tyr337; the rest of the molecular scaffold is oriented towards the main entrance of the enzyme, where it is notable that the amide moiety assists and stabilizes the binding through a polar network engaging the backbone of Phe295 and the carboxylic group of Asp74 with the role of a water molecule. Even more interestingly, the presence of the phenothiazine ring produces aromatic contacts with the Trp286 indole ring of the PAS and a critical hydrogen bond with the carbonyl moiety of Ser293, which might explain the low activity of the acetylated compound **13**.

It must be pointed out that a very similar binding is also achieved by the most potent AChE inhibitor of this series (**12d**) proving that the elongation of the carbon atom linker together with the presence of a piperazine instead of piperidine nucleus does not alter the binding mode, whose pattern fully resembles the interaction motif of donepezil reported by Gerlits et al. [35] (see Figure S3). As added value to these results, docking scores reported in Table 5 show that **11d** and **12d** are almost comparable in terms of binding energy, ligand efficacy and molecular similarity with donepezil, explaining their ability to efficiently inhibit AChE activity, and also that a small and electron withdrawing substitutions in *ortho* position of the benzyl moiety is needed to better close up the binding in the CAS portion of the enzyme.

**Figure 4:**
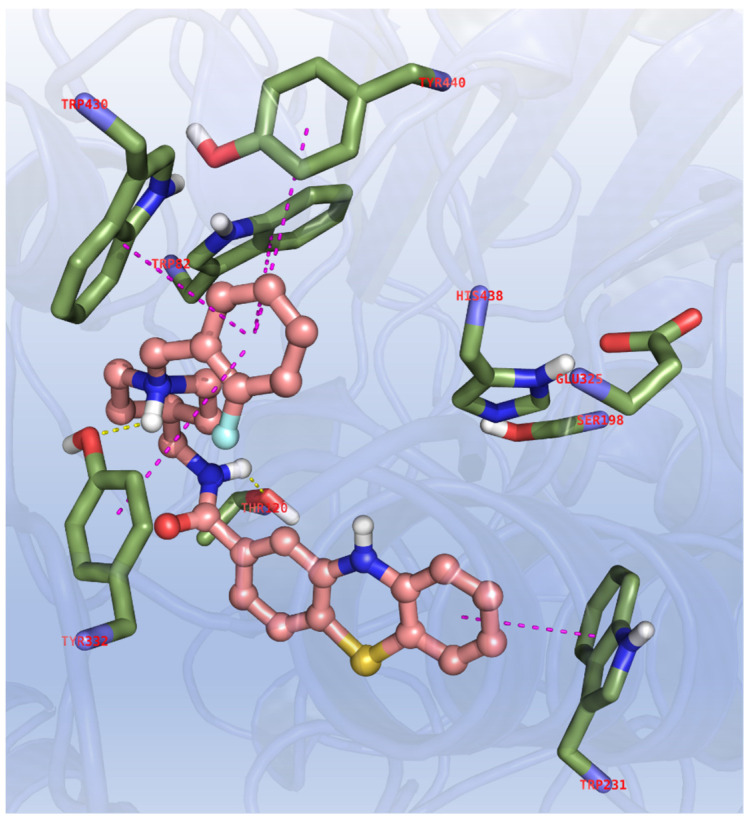
Binding mode for **11d** to the BChE active site. In the interaction pattern scheme hydrogen bonds and π-π stackings are depicted in yellow and magenta, respectively.

Binding of our compounds to BChE was also investigated, however a strict comparison of the interaction pattern previously observed in the **11d**/AChE complex cannot be applied due to relevant differences in the amino acid residues and accessible surface of the active site of the two cholinesterases. Supporting this concern, a curved conformation is mandatory for anchoring a binder and efficiently inhibit BChE, as observed in more than one X-ray complex of this enzyme; this is indeed notable in a recent crystallographic structure of a potent tacrine-methylanacardate hybrid inhibitor (**TKN**) active at the hBChE site, PDB code: 7BGC [52] that we decided to exploit in dockings of our phenothiazines to this enzyme. Thus, in this stage we observed that the molecular bundle of **11d** is deeply buried in the core of the catalytic site, with the benzyl aromatic terminal pendant stitched to the cluster of aromatic residues comprising the CAS, namely Trp82, Trp430, Tyr440 and Tyr332, with the latter amino acid embracing the basic nitrogen with a charged reinforced hydrogen bond (see Figure 4).

An additional polar interaction is also gained by the amide moiety forming a similar bond with the hydroxy group of Thr120, while the phenothiazine motif is edge-to-face π-π stacked with Trp231. The same indications gained from the previously reported docking scores are indeed observed for BChE (see Table 6; **ESP** parameters: **FEB** < −8.50, **ΔE** < 2.00, **EFF** < −0.200, **SIM** > 0.450, **POP** > 20)

**Figure 5:**
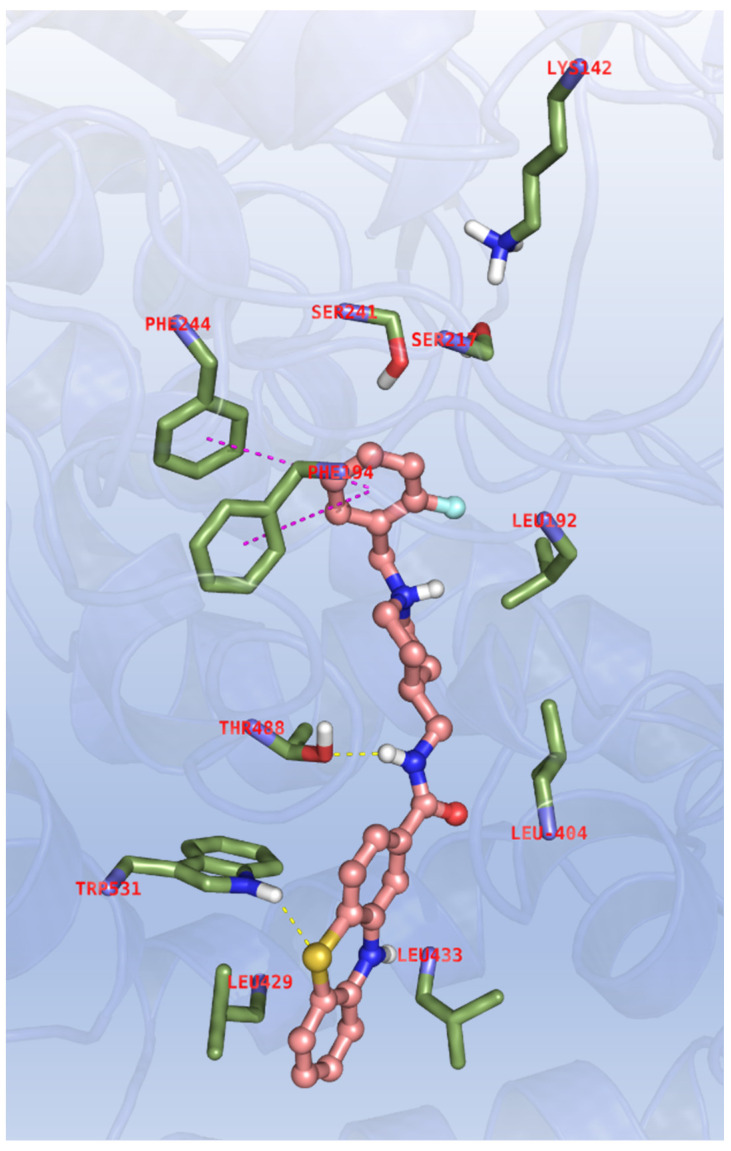
Binding mode for **11d** to the FAAH active site. In the interaction pattern scheme hydrogen bonds and π-π stackings are depicted in yellow and magenta, respectively.

In order to evaluate the multi-target profile of **11d** and **12d**, similar dockings were performed with FAAH, and therefore X-ray data of the enzyme in complex with the NSAID carprofen was selected (PDB code: 4DO3) [37]. For this enzyme the need for a different chemical cliché with respect to cholinesterases is evident from both primary sequence and structural features, because FAAH is a membrane bound enzyme and it also bears a different catalytic triad (Ser241, Ser217 and Lys142). Approaching the target, the binder enters the active site through an aromatic paddling gate comprising Phe432 and Trp531 and located at the boundary between the membrane-access and acyl chain-binding cavities pockets, forming the molecular recognition motif governing FAAH catalysis. It must be pointed out that phenothiazines and carprofen are significantly different molecular entities, and they might show different pharmacophoric features in the binding to FAAH. Therefore, docking models were interpreted through a comparison with the reference compound JZL195 (Figure S3) that is indeed more structurally related to the phenothiazine derivatives under investigation, and the binding mode of **11d** is reported in Figure 5.

The ligand is fully extended along the active site channel placing, in cooperation with Phe194 and Phe244, the *ortho*-fluoro substituted benzyl close to the catalytic triad, with the amide making hydrogen bond with the Thr488 side chain. The sulphur atom of phenothiazine ring interacts with the indole ring of Trp531, and additional hydrophobic contacts with Leu192, Leu404 and Leu433, located at the edge of the entrance cavity of FAAH, are observed. It is extremely interesting that this binding mode resembles more closely the one observed in the docking of **11d** with AChE, while being more different than the pose obtained with the same compound with BChE. Valuable scores are achieved in these dockings too (see Table 7; **ESP** parameters: **FEB** < −9.00, **ΔE** < 2.00, **EFF** < −0.250, **SIM** > 0.500, **POP** > 20)

### 3.4. ADME Properties

**Figure 6:**
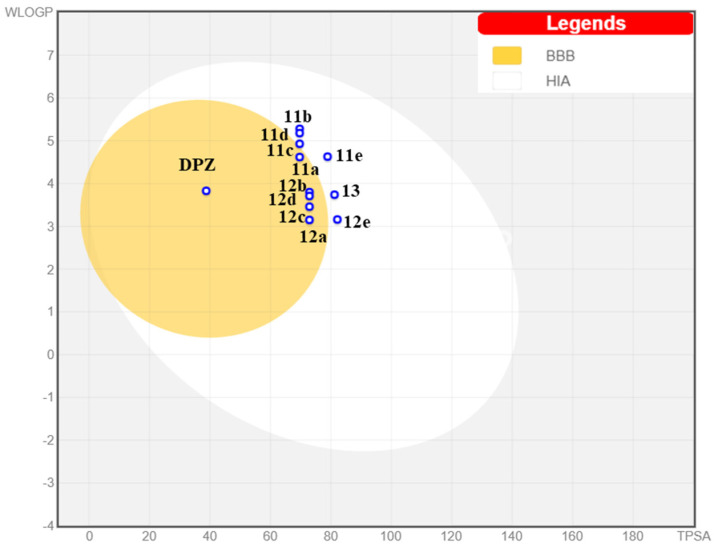
Prediction of pharmacokinetic properties of compounds **11a**–**e**, **12a–e** and **13** according to BoiledEgg. **BBB** = blood–brain barrier permeation; **HIA** = human intestinal absorption.

The Brain (or IntestinaL Estimate) permeation method [53] was used for drug-likeness estimation. As shown in Figure 6, all compounds in the series might be well absorbed in the gastrointestinal tract, so they spot in the white ellipse of the BOILED-Egg plot. Moreover, some of the studied compounds could potentially permeate the blood–brain barrier (yellow ellipse).

Further pharmacokinetic features were predicted by QikProp v.2.5 [46]. In particular, the compliance of the series was evaluated with “Lipinski’s rule of five”, as well as the molecular weight (MW), the oral absorption percentage, the CACO-2 cell permeability, the octanol-water partition coefficient (clog*P*) and the capacity to cross the BBB. These results are reported in Table S1.

The piperazine series of hybrids **12a–e** does not show violations of “Lipinski’s rule of five”, while piperidine hybrids **11a–e** (clog *P* > 5, in the range 5.342–5.71) and acetylated derivative **13** (MW > 5) violate only one rule. However, the violated parameter (molecular weight) is very close to the acceptable limit, although the violated parameters (clog *P* or MW) are relatively close to the acceptable limit. All compounds have 100% predicted oral absorption, and also the calculated octanol-water partition coefficient (clog*P*) and the BBB permeability coefficient (log BB) are both well within the acceptable ranges for drug-like compounds (−2 to −6.5 and −3 to −1.2, respectively). Concerning intestinal absorption, the shorter N-benzyl-piperidine group in **11a–e** causes great CACO-2 cell permeability, while the longer and more polar N-benzyl-piperazine moiety in **12a–e** and **13** leads to acceptable CACO-2 cell permeability values (88–135 nm/s). Besides, all compounds show CNS activity.

Finally, more strict physicochemical criteria for CNS drug candidates, such as MW < 450, clog *P* < 5, number of hydrogen bond donors HBD < 3, number of hydrogen bond acceptors HBA < 7 and polar surface area PSA < 60–70 [54] are not accomplished by compound **13** but compound **12a** fulfils them entirely. Moreover, several compounds have small discrepancies in terms of MW or clog *P*, such as **11d** (clog *P* = 5.52) and **12d** (MW = 462.58), which already proved to have multi-target biological activity.

## 4. Conclusions

In this study, a series of phenothiazine–donepezil hybrids were designed, synthesized, and assayed against different biological targets such as AChE, BChE and FAAH enzymes and Aβ aggregation. The experimental design was centered on the crucial role of the antioxidant activity, which was evaluated using different in vitro (DPPH and DCFDH-DA) assays. To rationalize the results, docking studies were then performed. Finally, in silico pharmacokinetic properties were also preliminarily evaluated, as well as the cytotoxicity on two different cell lines (HepG2 and SHSY-5Y).

Most compounds show interesting multi-target activity, in the low micromolar range, with good ADME predicted properties, also regarding the possibility of oral administration and ability to be active in CNS. Particularly attractive are the profiles of compounds **11d** and **12d**, with a fluorine atom in *ortho*-position on the benzyl group. In particular, the piperidine-based hybrid **11d** shows the best multi-target activity, being active in the range of concentration between 1.20 and 5.10 μM towards the targets tested except for the estimated ability to inhibit Aβ aggregation (43% at the concentration of 10 μM). The slightly lower-performing piperazine analogue **12d** is instead the most interesting Aβ aggregation inhibitor (I% = 60% at 10 μM), as well as a more selective AChE inhibitor (IC_50_ = 0.599 μM) and shows no cytotoxic effects on neuroblastoma cell lines at 24 h at 100 μM. In the case of **12d,** the activity window is somehow larger than for **11d** (between 0.599 and 14.6 μM, considering all assayed targets) but it is not too far from the ideal profile for a multi-target ligand.

Antioxidant assays highlight the importance of the non-substituted nitrogen atom of the phenothiazine moiety, which is confirmed as fundamental for this sort of activity. However, the results obtained with compound **13**, which is inactive in the DPPH test but is the best antioxidant in the in vitro assays on both cell lines, suggest the fascinating possibility to prepare effective pro-drugs in the near future using groups that are easily removable *in vivo*, a perspective that is further corroborated by the lack of cytotoxicity for this compound even at the relatively high dose of 100 μM. 

With this in mind, **11d** and **12d**, with their promising multi-functional potential, can be considered lead compounds for the development of novel multi-target drugs for the therapy of Alzheimer’s disease.

## Figures and Tables

**Table 1:** Biological activities of compounds **11a–e**, **12a–e** and **13** towards AChE, BChE, Aβ_1-40_ aggregation and FAAH.

Compound	X	n	R	R_1_	hAChE (IC_50_, µM) ^a^	hBChE (IC_50_, µM) ^a^	Aβ_1-40_ Aggregation (I% at 10 µM) ^a^	hFAAH (IC_50_, µM) ^a^
**11a**	CH	1	H	H	0.597 ± 0.064	2.16 ± 0.27	8% ± 5	22.6 ± 1.6
**11b**	CH	1	H	2-Cl	52% ± 1 ^b^	1.98 ± 0.12	20% ± 5	3.80 ± 0.90
**11c**	CH	1	H	2-CH_3_	44% ± 2 ^b^	1.47 ± 0.03	47% ± 6	3.08 ± 0.44
**11d**	CH	1	H	2-F	1.75 ± 0.11	1.20 ± 0.01	43% ± 3	5.06 ± 0.40
**11e**	CH	1	H	3-OCH_3_	22% ± 4 ^b^	47% ± 1 ^b^	24% ± 6	13.0 ± 3.4
**12a**	N	2	H	H	0.943 ± 0.046	2.02 ± 0.03	43% ± 6	41.4 ± 0.90
**12b**	N	2	H	2-Cl	1.85 ± 0.15	27% ± 2 ^b^	27% ± 6	6.31 ± 1.28
**12c**	N	2	H	2-CH_3_	56% ± 3 ^b^	2.50 ± 0.06	n.a.	7.33 ± 0.86
**12d**	N	2	H	2-F	0.599 ± 0.112	4.33 ± 0.19	60% ± 6	9.65 ± 2.45
**12e**	N	2	H	3-OCH_3_	48% ± 3 ^b^	6.70 ± 0.05	23% ± 6	8.65 ± 2.93
**13**	N	2	COCH_3_	2-Cl	48% ± 5 ^b^	15% ± 3 ^b^	n.a.	16.9 ± 4.4
**Donepezil**					0.017 ± 0.002	4.80 ± 1.00	14% ± 7	n.d.
**JZL195**					n.d.	n.d.	n.d.	0.019 ± 0.003

(^a^) Values are mean ± SEM; (^b^) I% at 10 µM; n.a. = not active, n.d. = not determined.

**Table 2:** Antioxidant potencies in the DCFH-DA assay for the set of compounds under study (**11a–e**, **12a–e**, and **13**) and quercetin.

Compound	DCFDH-DA IC_50_ ± SEM [μM] ^[a]^	
	HepG2	SHSY-5Y
**11 a**	8.31 ± 1.40	14.1 ± 0.60
**11b**	55.3 ± 1.10	1.32 ± 0.20
**11c**	17.6 ± 0.50	2.11 ± 0.40
**11d**	1.82 ± 0.60	1.43 ± 0.50
**11e**	3.21 ± 0.50	1.12 ± 0.90
**12a**	1.13 ± 0.41	2.01 ± 0.61
**12b**	18.7 ± 0.20	9.10 ± 1.42
**12c**	4.21 ± 0.81	11.3 ± 0.21
**12d**	14.6 ± 0.50	11.7 ± 1.11
**12e**	5.81 ± 0.90	1.93 ± 0.81
**13**	0.63 ± 0.10	1.22 ± 0.20
**Quercetin**	12.5 ± 0.41	5.61 ± 0.40

[a] Values are the mean of at least three determinations performed in triplicate.

**Table 3:** Free radical scavenging activity against DPPH for the studied set of compounds (**11a–e**, **12a–e**, and **13**) and gallic acid.

Compound	EC_50_ (μmol/μmol of DPPH ± SEM) ^[a]^
**11a**	0.082 ± 0.007
**11b**	0.139 ± 0.014
**11c**	0.117 ± 0.013
**11d**	0.126 ± 0.014
**11e**	0.104 ± 0.011
**12a**	0.182 ± 0.016
**12b**	0.122 ± 0.011
**12c**	0.145 ± 0.010
**12d**	0.231 ± 0.026
**12e**	0.104 ± 0.008
**13**	>1
**Gallic acid**	0.059 ± 0.003

[a] Values are the mean of at least three determinations performed in triplicate.

**Table 4:** IC_50_ of compounds under study (**11a–e**, **12a–e**, and **13**) in the MTT cytotoxicity assay on HepG2 and SHSY-5Y cells after 1 h or 24 h of exposure.

Compound	IC_50_ ± SEM [μM] ^[a]^			
	HepG2		SHSY-5Y	
	1 h	24 h	1 h	24 h
**11a**	>100	62.7 ± 0.70	>100	>100
**11b**	>100	27.7 ± 0.40	>100	21.2 ± 1.00
**11c**	>100	35.2 ± 1.10	>100	15.2 ± 1.20
**11d**	>100	41.0 ± 0.20	>100	9.33 ± 0.86
**11e**	>100	14.9 ± 0.61	>100	29.0 ± 1.10
**12a**	>100	28.8 ± 0.42	>100	>100
**12b**	>100	>100	>100	31.8 ± 2.0
**12c**	>100	>100	>100	>100
**12d**	>100	42.9 ± 1.81	>100	>100
**12e**	>100	39.4 ± 0.90	>100	12.1 ± 1.41
**13**	>100	>100	>100	55.3 ± 1.22

[a] Values are the mean of at least three determinations performed in triplicate.

**Table 5:** Docking scores for compound **11d** and **12d** to AChE.

	FEB ^(a)^	ΔE ^(b)^	EFF ^(c)^	TAN ^(d)^	POP ^(e)^
**11d**	−11.39	0.66	−0.356	0.822	40/1000
**12d**	−10.75	0.65	−0.326	0.815	45/1000
**Donepezil**	−10.30	0.05	−0.382	1.279	470/100

(^a^) **FEB** Free Energy of Binding (^b^) **∆E** Energy difference between the selected pose and the relative global minimum (^c^) **EFF** Ligand efficacy (^d^) **TAN** Tanimoto_Combo similarity coefficient of with donepezil X-ray pose (^e^) **POP** Cluster members population.

**Table 6:** Docking scores for compound **11d** and **12d** to BChE.

	FEB ^(a)^	ΔE ^(b)^	EFF ^(c)^	TAN ^(d)^	POP ^(e)^
**11d**	−9.66	0.00	−0.302	0.475	20/1000
**12d**	−9.39	0.39	−0.285	0.532	65/1000
**TKN**	−8.65	0.25	−0.247	1.174	21/100

(^a^) **FEB** Free Energy of Binding (^b^) **∆E** Energy difference between the selected pose and the relative global minimum (^c^) **EFF** Ligand efficacy (^d^) **TAN** Tanimoto_Combo similarity coefficient of with TKN X-ray pose (^e^) **POP** Cluster members population.

**Table 7:** Docking scores for compound **11d** and **12d** to FAAH.

	FEB ^(a)^	ΔE ^(b)^	EFF ^(c)^	TAN ^(d)^	POP ^(e)^
**11d**	−9.07	1.03	−0.283	0.652	11/1000
**12d**	−9.39	0.39	−0.285	0.532	65/1000
**JZL195**	−10.22	1.02	–0.319	nd	20/100

(^a^) **FEB** Free Energy of Binding (^b^) **∆E** Energy difference between the selected pose and the relative global minimum (^c^) **EFF** Ligand efficacy (^d^) **TAN** Tanimoto_Combo similarity coefficient of with JZL195 binding pose (^e^) **POP** Cluster members population.

## Data Availability

All of the data is contained within the article and the supplementary materials.

## Supplementary Materials

The following supporting information can be downloaded at: https://www.mdpi.com/article/10.3390/antiox11091631/s1, Figure S1 NMR spectra of selected final compounds; Figure S2 Summary chart of biological activities of final compounds **11a–c**, **11e**, **12a–c**, **12e** and **13**; Figure S3 Overlay of the binding mode of **11d**, TKN and JZL to AChE, BChE and FAAH; Table S1: Physicochemical descriptors and ADME properties of tested compounds **11a–11e**, **12a–12e** and **13** calculated by QIKPROP v. 2.5.
