# Supplementary material for: Novel Phenothiazine/Donepezil-like Hybrids Endowed with Antioxidant Activity for a Multi-Target Approach to the Therapy of Alzheimer’s Disease
Source: Antioxidants (Basel). 2022 Aug 23;11(9):1631. doi: 10.3390/antiox11091631 (PMC9495854; doi:10.3390/antiox11091631)
Supplement: Supplementary file 1 [file antioxidants-11-01631-s001.zip › antioxidants-1857993-supplementary.pdf]

Supplementary material

Novel Phenothiazine/Donepezil-like Hybrids Endowed with Antioxidant Activity for a Multi-Target Approach to the Therapy of Alzheimer's Disease

Alessia Carocci [1,*], Alexia Barbarossa [1,†], Rosalba Leuci [1,†], Antonio Carrieri [1], Leonardo Brunetti [1], Antonio Laghezza [1], Marco Catto [1], Francesco Limongelli [1], Sílvia Chaves [2], Paolo Tortorella [1], Cosimo Damiano Altomare [1], Maria Amélia Santos [2], Fulvio Loiodice [1] and Luca Piemontese [1,*]

[1] *Department of Pharmacy—Pharmaceutical Sciences, University of Bari Aldo Moro, via E. Orabona 4, 70125 Bari, Italy*

[2] *Centro de Química Estrutural, Institute of Molecular Sciences, Departamento de Engenharia Química, Instituto Superior Técnico, Universidade de Lisboa, Av. Rovisco Pais 1, 1049-001 Lisboa, Portugal*

* Corresponding authors: alessia.carocci@uniba.it; luca.piemontese@uniba.it
† These authors contributed equally to this work.

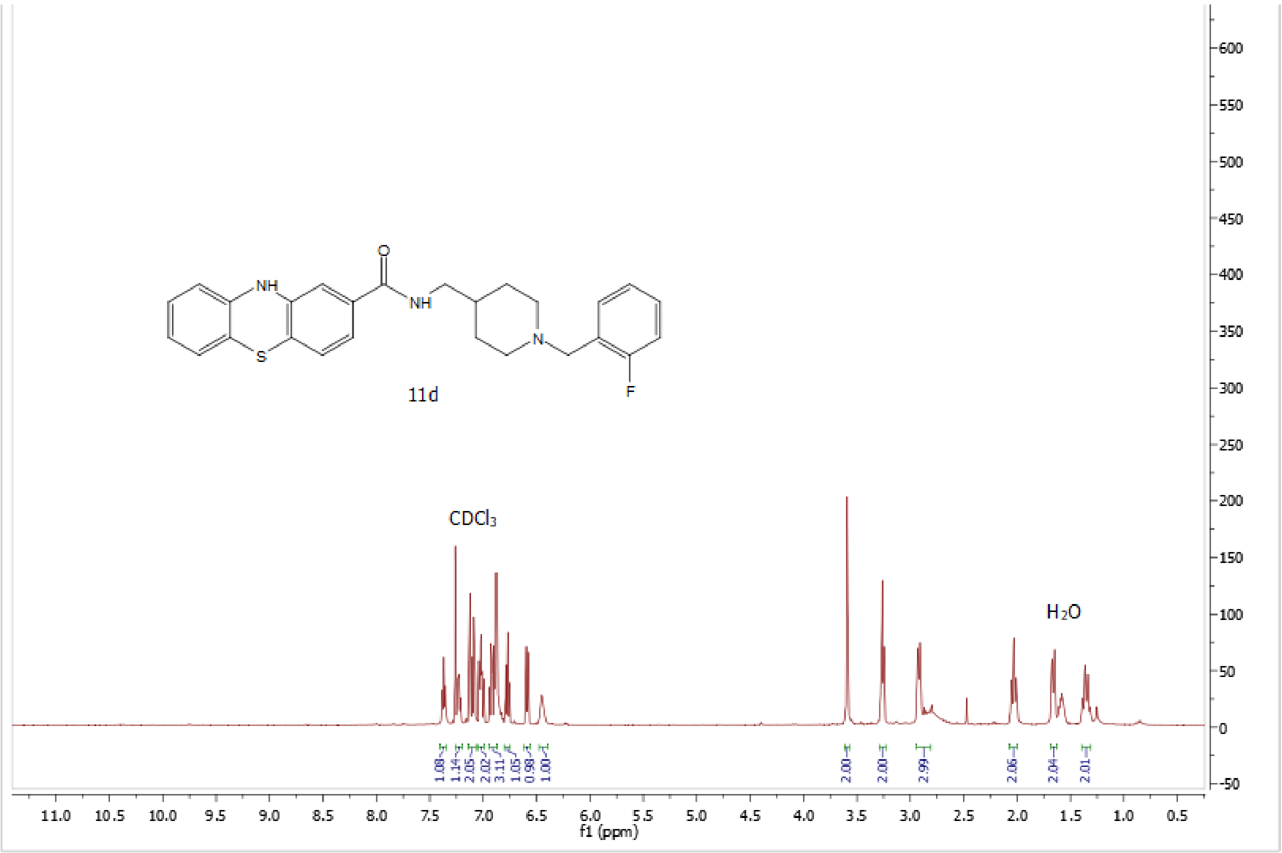
11d
CDCl3
H2O
f1 (ppm)

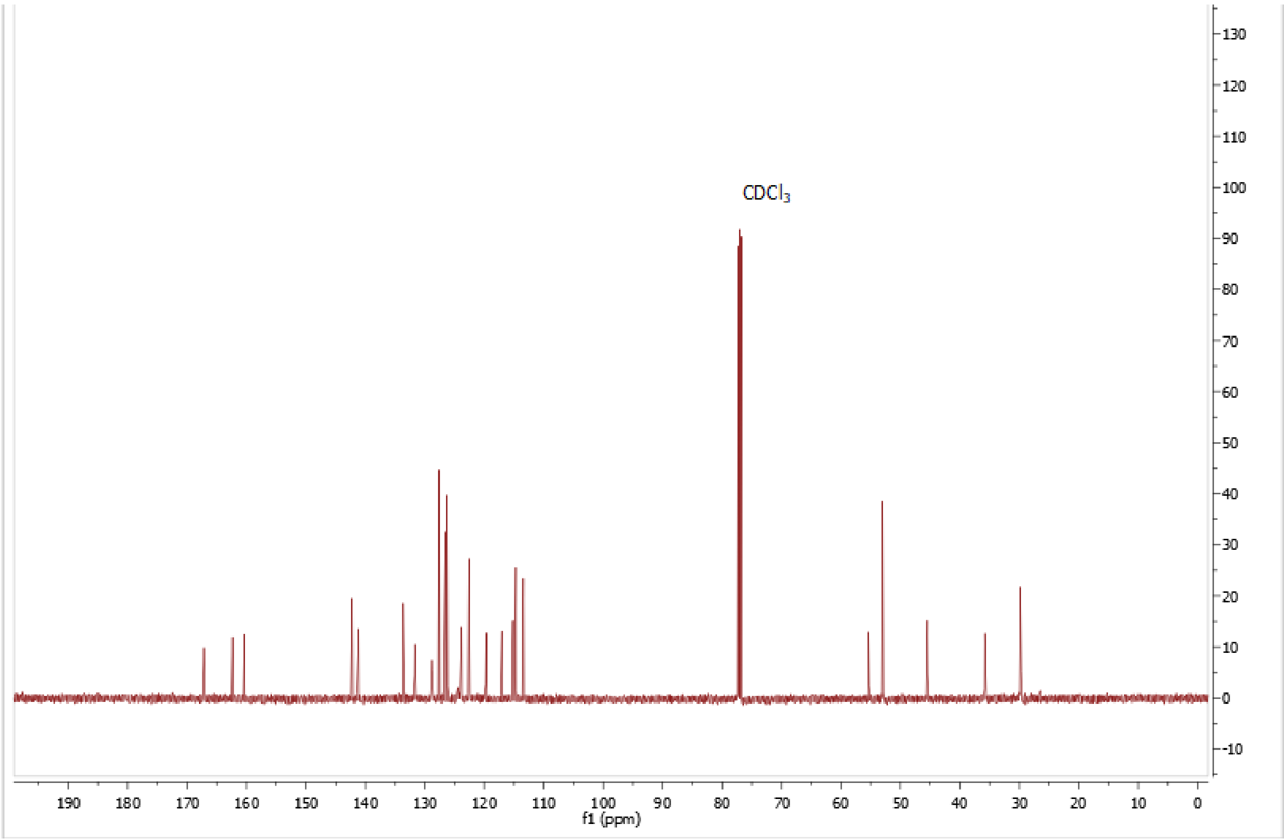
CDCl3
f1 (ppm)

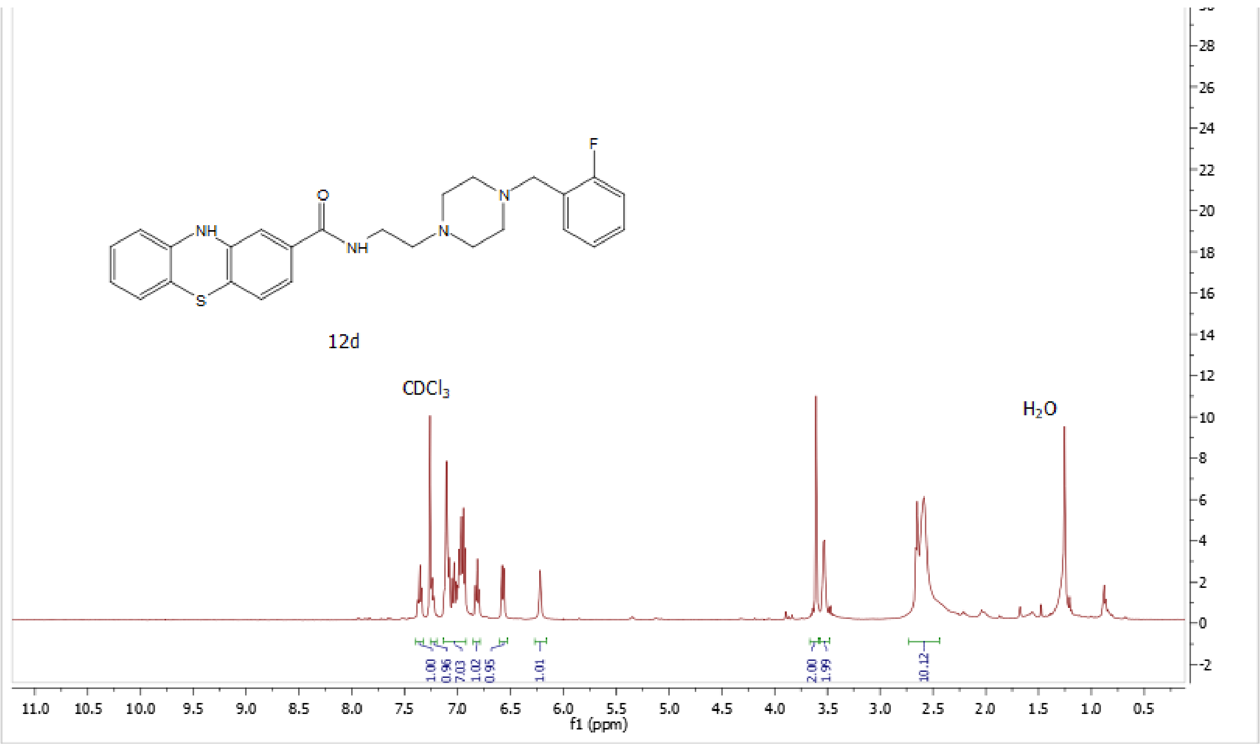

NH
O
NH
N
N
F
S
12d
CDCl3
H2O
f1 (ppm)

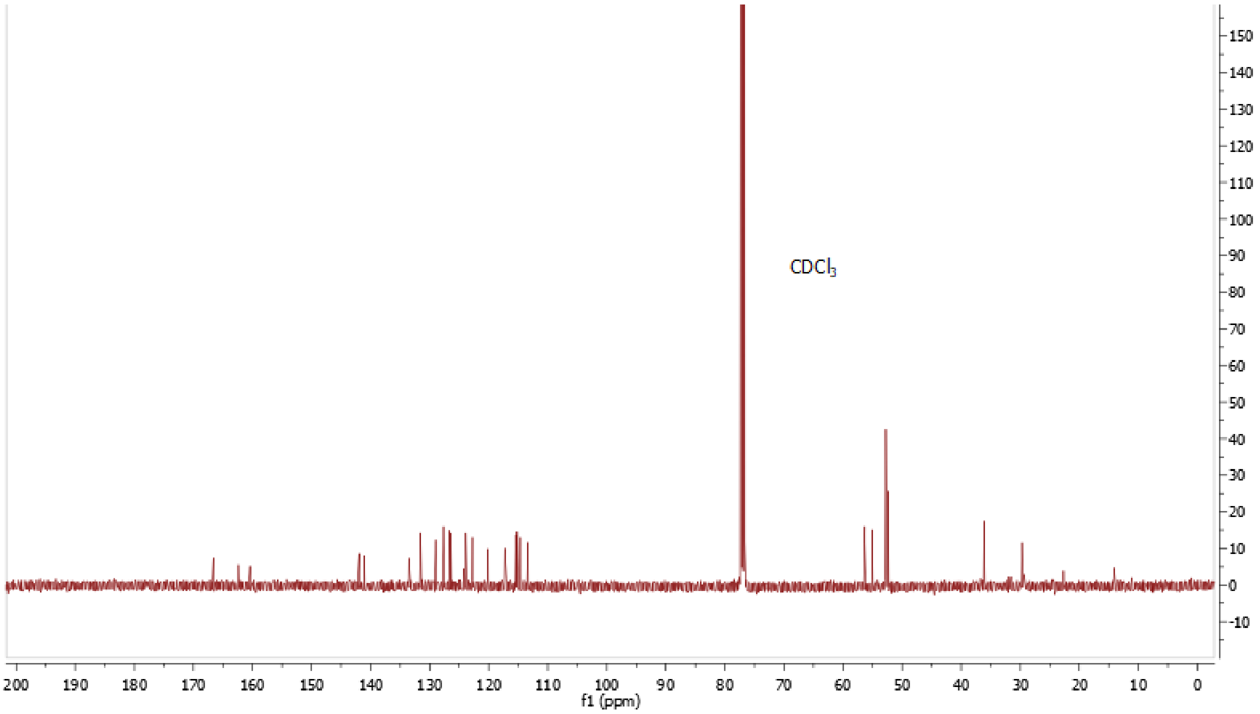

CDCl3
f1 (ppm)

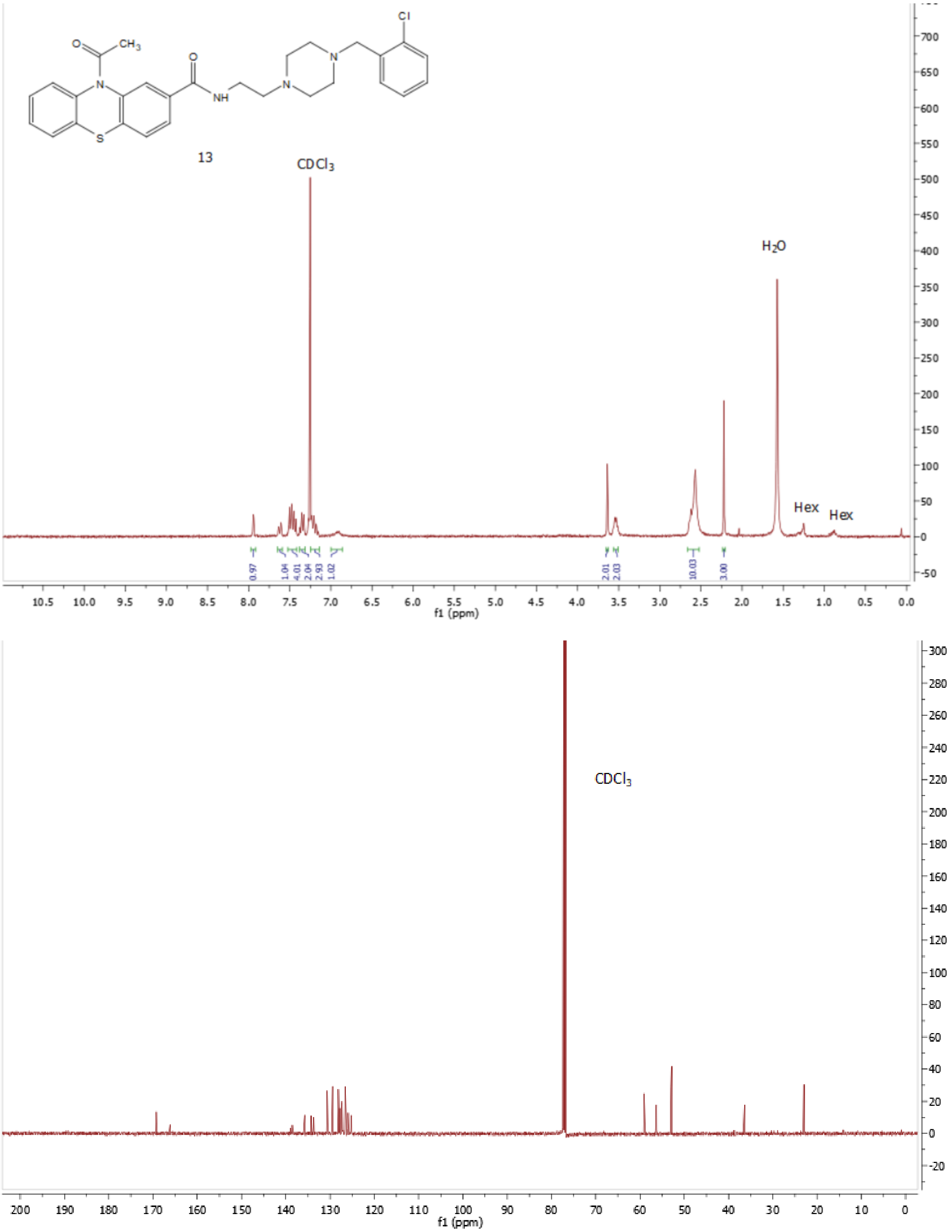

Figure S1 NMR spectra of selected compounds.

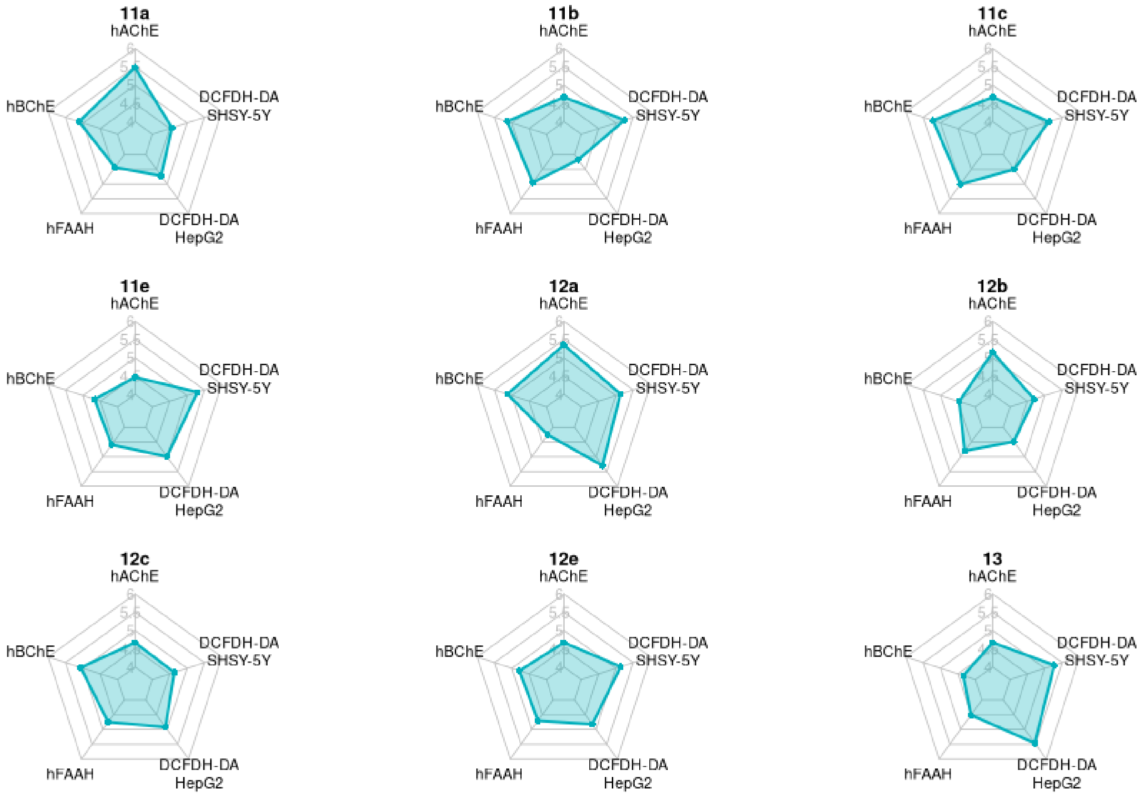

Figure S2. Summary chart representing the biological activities of compounds **11a–c**, **11e**, **12a–c**, **12e** and **13**. Data are reported as pIC_{50}. If IC_{50} was not evaluated the included data was estimated on the basis of the percentage of inhibition at 10 μM.

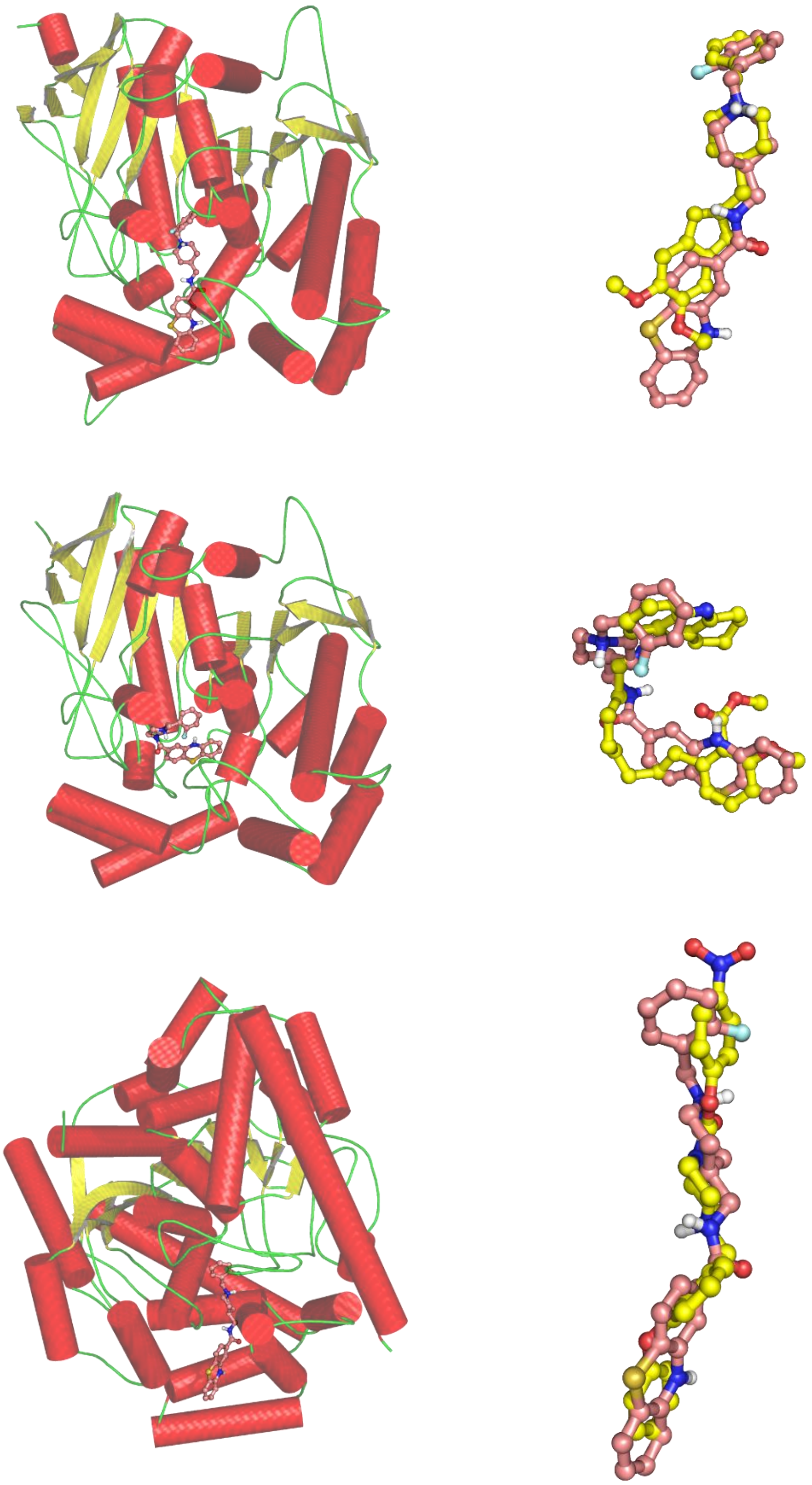

Figure S3 Overlay of the binding mode of **11d** (salmon carbons) and donepezil, TKN and JZL (yellow carbons) to AChE (top), BChE (center) and FAAH (bottom), respectively.

ADME prediction

Table S1. Physicochemical descriptors and ADME properties of tested compounds **11a-11e**, **12a-12e** and **13** calculated by QIKPROP v. 2.5.

Compound	MW[a]	clog *P*[b]	log BB[c]	P_{Caco-2}[d]	Oral Absorp.[e]	CNS act.[f]	Violations Rule of 5[g]	HBD[h]	HBA[i]	PSA[j]
**11a**	429.58	5.342	-0.015	552	100	+	1	2.0	5.0	53.49
**11b**	464.02	5.71	0.127	571	100	+	1	2.0	5.0	52.01
**11c**	443.61	5.58	0.008	573	100	+	1	2.0	5.0	51.72
**11d**	447.57	5.50	0.070	562	100	+	1	2.0	5.0	52.97
**11e**	459.60	5.44	-0.086	555	100	+	1	2.0	5.75	61.30
**12a**	444.59	4.27	0.288	135	100	+	0	2.0	7.0	59.22
**12b**	479.04	4.61	0.407	134	100	+	0	2.0	7.0	59.21
**12c**	458.62	4.49	0.288	135	100	+	0	2.0	7.0	59.02
**12d**	462.58	4.42	0.360	135	100	+	0	2.0	7.0	59.21
**12e**	474.62	4.36	0.217	135	100	+	0	2.0	7.75	67.12
**13**	521.08	3.91	0.230	88	100	+	1	1.0	9.50	71.81

[a](Acceptable <500); [b] Predicted octanol/water partition coefficient log *P* (acceptable range − 2.0 to 6.5); [c] Predicted BBB permeability (acceptable range − 3 to 1.2); [d] Predicted Caco-2 cell permeability in nm/s (acceptable range: < 25 is poor and > 500 is great); [e] Percentage of human oral absorption (acceptable range: < 25% is poor and > 80% is high); [f] qualitative CNS activity parameter from (--) inactive, (++) active; [g] Number of violations of Lipinski's rule of five; [h] number of hydrogen bond donors HBD; [i] number of hydrogen bond acceptors HBA; [j] polar surface area PSA.
